# Verteporfin-loaded hydrogel targeting YAP-mediated MDSCs recruitment for the treatment of residual tumors after incomplete radiofrequency ablation

**DOI:** 10.7150/thno.119377

**Published:** 2026-01-01

**Authors:** Jiawen Chen, Junfeng Liu, Xiaoting Zhang, Xi Li, Jinming Fan, Shengchao Zhao, Junbin Liu, Bin Zhou, Ke Zhang

**Affiliations:** 1 Center of Interventional Medicine, The Fifth Affiliated Hospital of Sun Yat-sen University, Zhuhai, Guangdong Province, 519000, China.; 2 Center of Cerebrovascular Disease, The Fifth Affiliated Hospital of Sun Yat-sen University, Zhuhai, Guangdong Province, 519000, China.; 3 Guangdong Provincial Engineering Research Center of Molecular Imaging, The Fifth Affiliated Hospital of Sun Yat-sen University, Zhuhai, Guangdong Province, 519000, China.; 4 Guangdong-Hong Kong-Macao University Joint Laboratory of Interventional Medicine, The Fifth Affiliated Hospital of Sun Yat-sen University, Zhuhai, Guangdong Province 519000, China.; 5 Guangzhou University of Chinese Medicine-Shenzhen Hospital, Shenzhen, Guangdong Province, 518000, China.; 6 Department of Interventional Therapy, Sichuan Clinical Research Center for Cancer, Sichuan Cancer Hospital & Institute, Sichuan Cancer Center, Affiliated Cancer Hospital of University of Electronic Science and Technology of China, Chengdu Province, 610041, China.

**Keywords:** hepatocellular carcinoma, radiofrequency ablation, yes-associated protein, verteprofin, hydrogel

## Abstract

**Background:** Hepatocellular carcinoma (HCC), the major form of primary liver cancer, contributes markedly to cancer-related mortality worldwide and remains a serious global health concern, particularly affecting individuals with underlying chronic liver disorders. In hepatocellular carcinoma, insufficient radiofrequency ablation (iRFA) has been reported to drive local tumor relapse and distant spread, possibly by aggravating the immunosuppressive features of the tumor microenvironment. The present work seeks to clarify the underlying pathways driving the development of an immunosuppressive milieu after RFA and to identify potential therapeutic approaches to counteract this process.

**Methods:** An injectable hydrogel composed of quaternized chitosan (QCS) and tannic acid (TA) was constructed to encapsulate verteporfin (VP), a well-established photosensitizer that has been clinically applied for treating neovascular retinal disorders such as age-related macular disease. Beyond its ophthalmologic application, VP has recently been reported to display anti-tumor activity through inhibition of oncogenic regulators such as Yes-associated protein (YAP), indicating its potential utility in cancer therapy. This hydrogel formulation is designed to target residual tumor tissue post-RFA, providing localized delivery and sustained release of VP to enhance anti-tumor immune responses.

**Results:** Our findings identified YAP activation as a critical mediator of immunosuppression in residual tumors following RFA. Pharmacological inhibition of YAP significantly reduced the infiltration of myeloid-derived suppressor cells (MDSCs) and effectively reversed the immunosuppressive microenvironment conditions. Furthermore, the QCS/TA hydrogel enabled sustained local release of VP, resulting in enhanced antitumor immune responses via MDSC suppression. When administered as an adjuvant therapy following suboptimal RFA, the hydrogel markedly inhibited the progression of residual tumors, highlighting its therapeutic potential in improving post-RFA outcomes.

**Conclusion:** Collectively, our data suggest YAP pathway inhibition as a promising immunomodulatory strategy to complement RFA in HCC management. This work demonstrates that the QCS/TA hydrogel-based delivery system can remodel the tumor immune milieu to overcome immunosuppression and delay post-ablation tumor recurrence, supporting its potential as a translational drug delivery strategy.

## Introduction

Globally, hepatocellular carcinoma (HCC) represents a significant oncological challenge, ranking among the top causes of cancer-related mortality, particularly in populations affected by chronic liver disorders. Among various treatment modalities, radiofrequency thermal ablation (RFA) has become a widely utilized, minimally invasive technique for managing early-stage HCC, offering favorable outcomes and lower morbidity compared to surgical resection 1. Recent years have witnessed remarkable clinical advances in cancer management, ranging from surgical precision techniques to molecularly targeted therapies. For example, the use of sentinel node mapping has become a highly accurate and minimally invasive technique for evaluating malignancies in the oral and pharyngeal regions 2, whereas combining lymphaticovenular bypass with nodal transplantation offers encouraging potential for managing lymphedema secondary to breast cancer. 3. Moreover, advances in nanocarrier-mediated drug delivery and gasotransmitter-related therapeutic modalities have introduced novel avenues for improving treatment precision and remodeling the tumor microenvironment 4,5. Despite these advances, the recurrence rate of HCC following RFA remains high, exceeding 70% within 5 years. This limitation becomes particularly evident in tumors situated near critical organs or major vessels, in those with irregular margins, or in lesions exceeding 3 cm in diameter, all of which are predisposed to incomplete radiofrequency ablation (iRFA) 6-12. However, insufficient thermal coverage or tumor proximity to vital structures often leads to iRFA, which can lead to residual tumor cells that not only survive but also exhibit enhanced aggressiveness, ultimately contributing to an elevated incidence of local recurrence and distant metastasis 13-16. These constraints markedly hinder the broader clinical application of RFA, whereas the mechanistic basis behind these limitations has yet to be fully elucidated.

Recent evidence indicates that alterations within the tumor immune microenvironment are pivotal to this process. Accumulating evidence indicates that iRFA-induced residual tumors foster a profoundly immunosuppressive microenvironment, which hampers antitumor immune responses and facilitates tumor progression. Furthermore, iRFA has been reported to promote the infiltration of suppressive immune cells, including tumor-associated macrophages (TAMs) and myeloid-derived suppressor cells (MDSCs), thereby facilitating immune escape of residual tumor cells. In this study, we observed that iRFA reshapes the tumor microenvironment toward an immunosuppressive phenotype, marked by elevated MDSC infiltration and a diminished population of CD8⁺ T cells. MDSCs, which exert strong inhibitory effects on anti-tumor immunity, were markedly enriched in the post-ablation regions and are believed to contribute to tumor immune evasion and therapeutic resistance. Despite this understanding, the molecular mechanisms governing immune suppression in the post-RFA context remain incompletely defined. Therefore, targeting these key mechanisms and reactivating anti-tumor immunity may represent an effective strategy for treating residual tumors and overcoming the limitations of RFA therapy 7,17,18.

Yes-associated protein (YAP), a key downstream mediator of the Hippo pathway, function as a nuclear transcriptional regulator that critically regulates tumor initiation and progression 19. Notably, YAP activity has been reported to be induced by heat shock 20. Once activated, dephosphorylated YAP translocated to the nucleus, where it exerts pro-oncogenic effects 21-23. Furthermore, studies have highlighted the close connection between YAP and the tumor immune microenvironment. In prostate cancer 24, lung adenocarcinoma 25, and colorectal cancer 26, YAP has been shown to recruit MDSCs by upregulating the expression of specific cytokines or chemokines. This cellular recruitment facilitates the establishment of an immunosuppressive tumor milieu that fosters cancer cell survival and progression.

Verteporfin (VP), a pharmacological YAP inhibitor, has been demonstrated to block YAP's oncogenic effects by disrupting its interaction with TEAD or enhancing its cytosolic retention via 14-3-3-mediated anchoring 27-31. Clinically, VP is extensively employed to manage ocular neovascular conditions, particularly age-related retinal degeneration, owing to its well-established safety record 30. In recent years, VP has attracted growing interest in oncology, with multiple preclinical studies and clinical trials evaluating its efficacy in various malignancies via YAP-targeted mechanisms 27,28,32,33. However, systemic delivery of verteporfin is limited by its inability to maintain adequate and prolonged drug levels within the tumor region 27,34,35 along with the potential for off-target systemic toxicity 36,37 when applied to iRFA residual lesions. Besides, it remains unclear whether targeting YAP with VP can effectively modulate the immunosuppressive microenvironment and inhibit residual tumor progression following iRFA. To bridge this knowledge gap, we explored the therapeutic efficacy of verteporfin (VP) in hepatocellular carcinoma models following incomplete ablation.

To address the limitations of current RFA-based therapies and improve clinical outcomes, we developed an injectable hydrogel system composed of quaternized chitosan (QCS) and tannic acid (TA), encapsulating the photosensitizer verteporfin (VP). This hydrogel was engineered to enable sustained local delivery of VP, aiming to suppress YAP signaling, mitigate MDSC-mediated immune suppression, and boost antitumor immunity in residual tumors. Compared with unmodified chitosan (CS), QCS was selected for its permanent positive charge and excellent solubility under physiological conditions, which ensures stable injectability and reproducible gelation *in vivo*. Moreover, the stronger electrostatic and hydrogen-bonding interactions between QCS and TA enable rapid and controllable complexation, forming hydrogels with desirable self-healing and tissue-adhesive properties. These advantages make QCS/TA hydrogels more suitable for localized, sustained drug delivery applications in tumor therapy.

**Figure 1:**
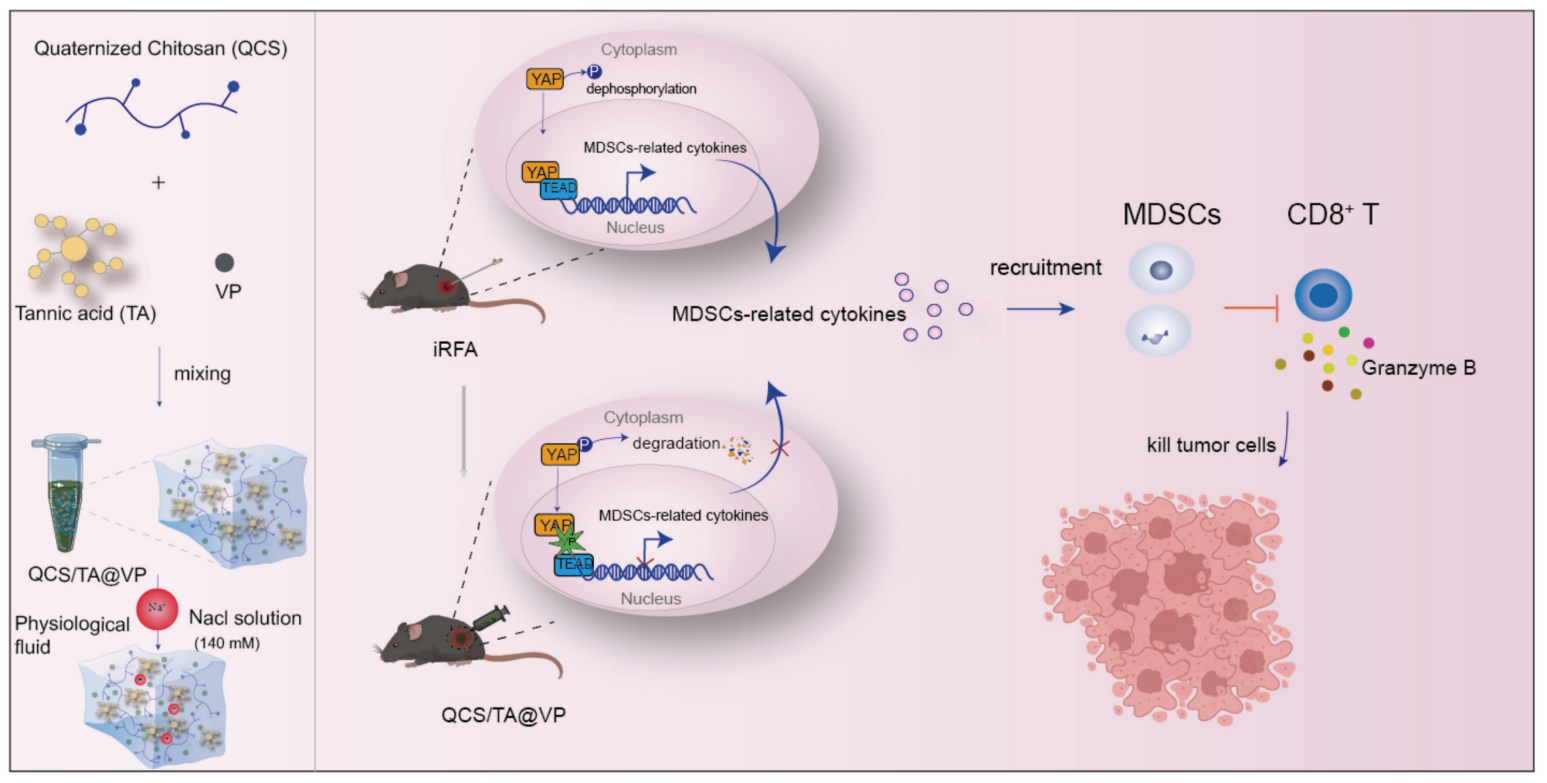
**Schematic showing QCS/TA@VP-enhanced RFA efficacy in HCC through immune microenvironment reprogramming.** iRFA typically induces an immunosuppressive tumor microenvironment. However, injection of QCS/TA@VP into the residual tumor cavity post-RFA counteracts this by reducing MDSCs infiltration and increasing CTLs infiltration, thereby enhancing the anti-tumor immune response.

This work elucidates the involvement of YAP in immune landscape remodeling of residual tumors post-iRFA and highlights the QCS/TA-VP hydrogel as a promising adjuvant intervention. Treatment with the YAP inhibitor verteporfin led to a reduction in MDSCs infiltration and significant inhibition of residual tumors following iRFA (Figure 1). Our findings offer mechanistic insights into post-ablation immune dynamics and provide a novel, clinically translatable approach to prevent tumor recurrence and enhance immunotherapeutic responses in HCC.

## Methods

### Reagents and equipment

Verteporfin (S1786) was sourced from Selleck Chemicals (USA). Antibodies against YAP (Cat#14074S) and phosphorylated YAP (p-YAP, Cat#13008T) were supplied by Cell Signaling Technology (CST), USA. Alexa Fluor® 488-labeled goat anti-rabbit IgG (ab150077) was purchased from Abcam (USA). Quaternized chitosan (QCS, S24030) was supplied by Shanghai Yuanye Biotechnology Co., Ltd. (China), and tannic acid (TA, T308008) was obtained from Aladdin (Shanghai, China).

### Cell lines and animals

Hepa1-6 and Hep3B cells lines were sourced from the ATCC (Manassas, VA, USA). Cells were maintained in Dulbecco's Modified Eagle Medium (DMEM) containing 10% FBS (MK1123, MIKX, China) and 1% penicillin-streptomycin at 37 °C in a humidified atmosphere containing 5% CO₂. Female C57BL/6 mice (4-6 weeks old) were abtained from the Laboratory Animal Center of Guangdong Province. All experimental procedures involving animals were approved by the Institutional Animal Care and Use Committee of the Fifth Affiliated Hospital of Sun Yat-sen University (Ethical reference number: 00482).

### Establishment of Yap1 knockout Hepa1-6 cell line

Lentiviral Yap1 knockdown was performed using three different shRNAs targeting mouse Yap1 (pLKO.1-U6-Mus-Yap1-sh1/sh2/sh3-EF1a-copGFP-T2A-puro; IGE Biotechnology Ltd., Guangzhou, China). Lentivirus was packaged and transduced into Hepa1-6 cells following standard protocols, and puromycin (2 μg/mL) was applied 48 h post-infection to selected successfully transduced cells.

### Real-time PCR subsequent to total RNA extraction

Total RNA was islolated from cultured cell and tissues specimens with the RNAiso Plus reagent (Vazyme Biotech, Nanjing, China). cDNA was synthesized from 1 μg of total RNA employing the HiScript III Reverse Transcriptase kit (Vazyme Biotech, Nanjing, China) according to the supplier's instructions. Quantitative PCR was carried out using ChamQ Universal SYBR qPCR Master Mix (Vazyme Biotech Co., Ltd., Nanjing, China) on a CFX96 Touch™ Real-Time PCR System (Bio-Rad Laboratories, Hercules, USA). Primer information (Tsingke Biotechnology Co., Ltd., Beijing, China) is listed in Table S1. Relative gene expression levels were normalized to β-actin or GAPDH and calculated via the 2⁻(ΔΔCt) method.

### Western blot analysis

Total protein extracts (40-60 μg per sample) were separated on 10% polyacrylamide gels (Bio-Rad Laboratories, USA) and transferred to nitrocellulose membranes (Pall, MI, USA). Membranes were blocked with 5% skimmed milk in TBST for 1 h at room temperature and subsequently incubated with primary antibodies diluted in 1% casein/TBST overnight at 4 °C. After thorough rinsing, membranes were treated with infrared dye-labeled secondary antibodies (1:5000) for 1 h. Protein signals were visualized and quantified using an iBright™ CL750 imaging system (Thermo Fisher Scientific, USA).

### Immunohistochemistry (IHC) staining

Tissue specimens were immersed in 4% paraformaldehyde, dehydrated through graded ethanol solutions, and embedded in paraffin wax. Paraffin-embedded tumor sections were prepared and subjected to immunohistochemical staining using an anti-YAP antibody (CST, USA) following the manufacturer's instructions. We evaluated the DAB staining scores using Image J software and the IHC Profiler plugin 38. The scoring of the areas was defined as follows: 4 for strong positive, 3 for moderate positive, 2 for weak positive, and 1 for negative zones. The final score was calculated according to the algebraic formula:



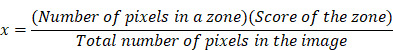



### Immunofluorescence

Following fixation in 4% paraformaldehyde for 20 min at ambient temperature, the cells were subsequently treated with PBS supplemented with 10% goat serum (Boster Biological Technology, Wuhan, China; Cat# AR1009) and 0.1% Triton X-100 for 30 min to achieve permeabilization and blocking. After exposure to the primary antibodies, cells were treated with fluorescent secondary antibodies, and images were perfromed using a Zeiss LSM 880 confocal laser scaning microscope. We assessed the nuclear/cytoplasmic fluorescence intensity ratio using Image J following the method described in the literature 39.

### Preparation of hydrogel

QCS and TA powders were each dissolved in deionized water to obtain 4% (w/v) solutions, which were then combined at a ratio of 2:3.

### Drug release tests

VP (3mg) was loaded into 1 mL of QCS/TA hydrogel and left to equilibrate at ambient temperature for 24 h. Subsequently, 500 μL of the prepared QCS/TA@VP hydrogel was placed in 2 mL of PBS to evaluate drug release. At predetermined time points, 1 μL of the supernatant was collected, appropriately diluted, and its absorbance at 430 nm was measured using a UV-Vis spectrophotometer, from which the cumulative VP release was quantified.

### Flow cytometry analysis

At day 7 following treatment, single-cell suspensions were generated from tumor and spleen specimens through mechanical dissociation. To block nonspecific Fc receptor binding, the cells were first incubated with anti-CD16/32 antibodies (Elabscience, E-AB-F0997A) and subsequently labeled with the following fluorophore-labeled antibodies following the supplier's protocol: Percp/Cy5.5-anti-CD45 (BioLegend, 103132), FITC-anti-CD11b (BioLegend, 101205), PE/Cy7-anti-Gr-1 (BioLegend, 108415), BV421-anti-F4/80 (BioLegend, 123137), BV605-anti-CD86 (BioLegend, 105037), PE/Cy7-anti-CD206 (BioLegend, 141719), APC/Cy7-anti-CD3 (BioLegend, 100222), BV605-anti-CD4 (BioLegend, 100451), FITC-anti-CD8 (BioLegend, 553030), and PE-anti-Granzyme B (BioLegend, 372207). Flow cytometric analysis was carried out with a CytoFLEX LX flow cytometer (Beckman Coulter, USA), acquiring 1.0 × 10⁴ CD45⁺ events per sample. Subsequent data processing and analysis were performed with FlowJo software (v10.8.1). The gating scheme is presented in Figure S4 of the Supplementary Information.

### *In vivo* experiment

A subcutaneous Hepa1-6 tumor model was generated by inoculating 1 × 10⁶ Hepa1-6 cells into the right dorsal region of C57BL/6 female mice. Therapeutic administration commenced once the tumor size grew to around 300 mm³. To simulate sublethal ablation conditions *in vivo*, we established an iRFA model in hepatocellular carcinoma-bearing mice, as previously described 40. Residual tumor viability after iRFA was confirmed by IVIS imaging, which showed detectable bioluminescence signal in the ablated region. Furthermore, histopathological analysis revealed partial tumor necrosis post-iRFA, in contrast to the fully viable tumors in the untreated HCC group (Figure S1). These results collectively confirm the successful induction of a sublethal ablation model with preserved tumor burden.

To explore the *in vivo* therapeutic potential of QCS/TA@VP, four randomized mouse groups (n = 6 per group) were established for treatment evaluation. The experimental cohorts included iRFA alone, iRFA combined with blank QCS/TA hydrogel, iRFA with free VP, and iRFA with QCS/TA@VP hydrogel. Following iRFA treatment, 100 μL of PBS, blank QCS/TA hydrogel, free VP, or QCS/TA@VP hydrogel was administered into the ablation cavity. The VP dosage for each mouse corresponded to 100 mg/kg. Tumor progression was recorded, and volumes were determined using the equation: volume= width^2^ × length/2 40,41.

### Statistical analysis

All statistical processing was conducted using GraphPad Prism software (v10.0). Quantitative data are presented as the mean ± standard error of the mean (SEM), and N denotes the number of independent biological replicates. Differences between two groups were assessed using a two-sided Student's *t*-test, while multiple comparisons were performed by one-way ANOVA followed by Tukey's multiple comparison test. Significance levels were defined as not significant (ns) or statistically significant at *P* < 0.05 (*),* P* < 0.01(**),* P* < 0.001(***), and *P* < 0.0001 (****).

## Results

### Activation of YAP in residual tumor after iRFA

**Figure 2:**
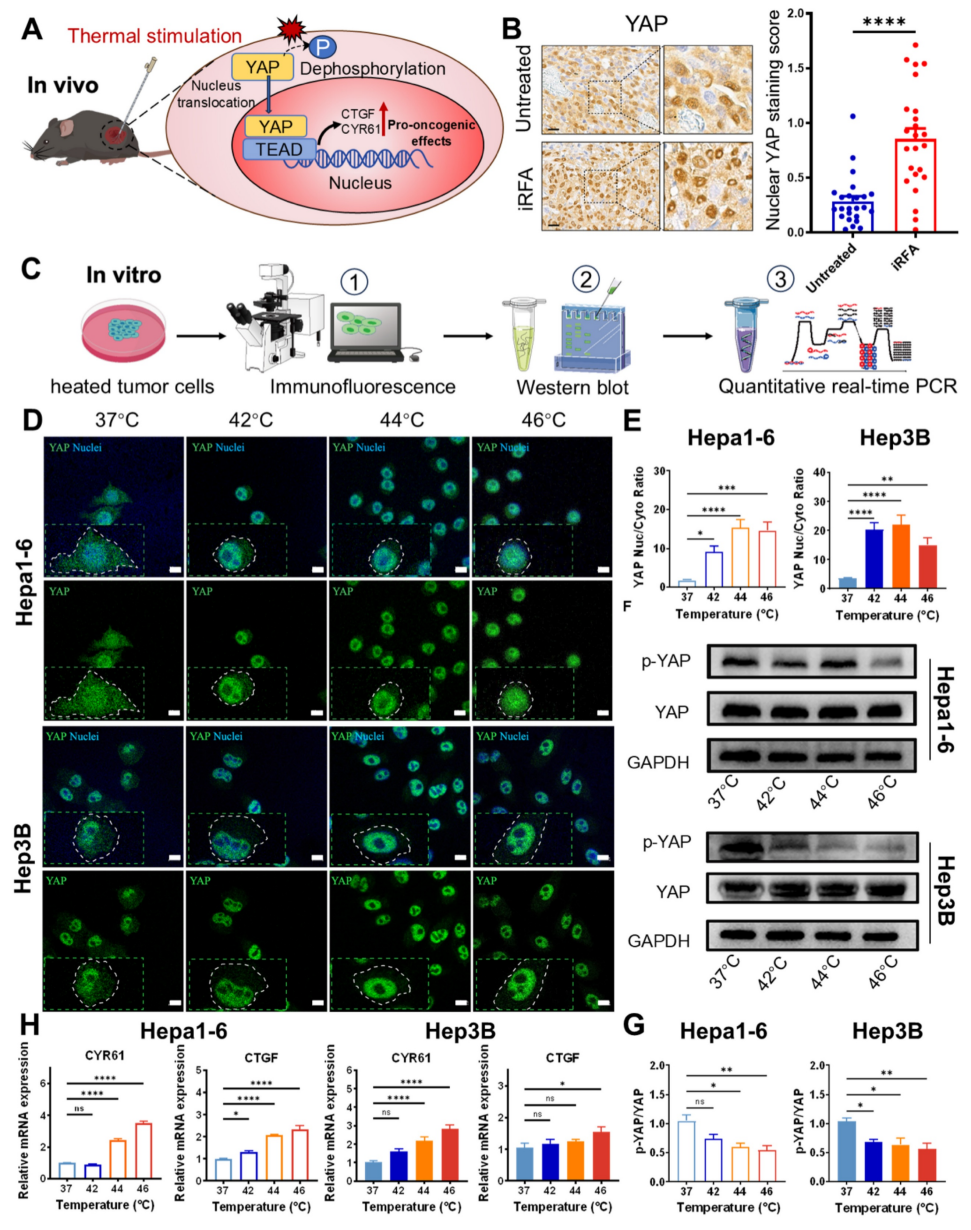
** Activation of YAP in residual tumor after iRFA.** (A) Schematic illustration of YAP activation following iRFA. (B) Immunohistochemical (IHC) staining of nuclear YAP (left) expression and quantitative YAP staining score (right) in iRFA-treated and nontreated tumor tissues. Scale bar = 20 μm. For each animal, six non-overlapping fields were randomly selected for analysis (n = 4). (C) Schematic of *in vitro* experiments evaluating temperature-induced YAP activation. (D) Immunofluorescence staining images of the Hepa1-6 and Hep3B cells and their YAP nuclear-cytoplasmic ratio (E) treated with various temperature. Cells were stained for YAP (green), and nuclei were labeled with DAPI (blue). Scale bar = 10 μm. (F) Western blot analysis of total YAP and phosphorylated YAP (p-YAP) protein levels in Hepa1-6 and Hep3B cells exposed to varying temperatures (n = 3). (G) Quantitative densitometric analysis of YAP and p-YAP protein expression. (H) Content of CYR61 and CTGF in Hepa1-6 and Hep3B cells treated with various temperature (n = 3). Statistical significance: ns, not significant; *P < 0.05; **P < 0.01; ***P < 0.001; ****P < 0.0001,

YAP functions as the central regulator of the heat shock response by orchestrating transcriptional programs that enhance stress adaption and promote cell survival under thermal challenge 20. Following thermal stimulation, dephosphorylated YAP undergoes nuclear translocation and acts as a transcriptional activator, driving oncogenic programs via upregulation of downstream effectors including CTGF and CYR61 (Figure 2A) 42,43.

Clinical evidence from a meta-analysis by Lin *et al.* (n = 391 HCC cases, 334 controls) established significant correlations between YAP overexpression and aggressive tumor characteristics: vascular invasion (OR = 2.21, 95% CI 1.64-2.97, *P* < 0.00001), poor cellular differentiation (OR = 2.38, 95% CI 1.61-3.51, *P* < 0.00001), tumor size > 5.00 cm (OR = 2.52, 95%CI 1.75-3.62, *P* < 0.00001), and advanced TNM staging (OR = 0.44 for I+II vs. III+IV, 95% CI 0.28-0.75, *P* = 0.00003) 44. To further evaluate its prognostic value in advanced HCC, we performed subgroup analysis on T2-T4 stage patients from TCGA cohort. Kaplan-Meier analysis indicated that high YAP expression group (n = 71) had significantly lower 5-year overall survival rate (36.10%) compared to low expression group (n = 70, 42.80%), with log-rank *P* = 0.026 (Figure S2).

To investigate whether YAP contributes to residual tumor progression after iRFA, we treated the tumor in murine model with iRFA and collected residual tumor tissues. Immunohistochemical (IHC) staining documented a marked enhancement of nuclear YAP localization in iRFA-treated tumors compared to untreated controls. The iRFA-treated group exhibited elevated nuclear YAP staining compared to the controls. (*P* < 0.0001) (Figure 2B).

To systematically characterize YAP subcellular redistribution under thermal stress, we subjected murine Hepa1-6 and human Hep3B cell lines to controlled temperature treatments (e.g. 37, 42, 44 and 46 °C) (Figure 2C). Immunofluorescence (IF) staining revealed altered YAP subcellular localization following heat treatment. Compared untreated controls, heat-exposed cells exhibited reduced YAP fluorescence intensity in the cytoplasm alongside increased nuclear YAP accumulation (Figure 2D). Quantitative measurements revealed a statistically significant increase in the nuclear-to-cytoplasmic YAP ratio following heat treatment. At 46 °C, the ratio exhibited an 8.6-fold elevation in Hepa1-6 cells (P < 0.001) and a 4.3-fold rise in Hep3B cells (P < 0.01) relative to the control group (Figure 2E). These results confirming heat stress-induced YAP nuclear translocation *in vitro*. Consistently, Western blot (WB) results showed decreased levels of phosphorylated YAP (p-YAP) proteins in both cells post-heat treatment, with phospho-YAP (p-YAP) decreased by 46 ± 12% in Hepa1-6 and 44 ± 16% in Hep3B at 46 °C (Figure 2F and 2G). The shift in YAP subcellular localization paralleled its post-translational modification, demonstrating that thermal stress attenuates YAP phosphorylation.

Additionally, RT-qPCR analysis revealed that heat exposure at 46 °C significant upregulated CYR61 expression by 3.5 ± 0.3-fold in Hepa1-6 cells and 2.8 ± 0.8-fold in Hep3B cells, while CTGF expression increased by 2.3 ± 0.5-fold (Hepa1-6) and 1.5 ± 0.5-fold (Hep3B), These changes indicate enhanced transcriptional activity of YAP under thermal stress (Figure 2H). Notably, the threshold for significant YAP activation occurred between 42-46 °C, a temperature range particularly relevant to clinical iRFA applications as it corresponds to typical periablational zone conditions. Collectively, these findings indicate that iRFA triggers YAP activation through dephosphorylation and its relocation to the nucleus, subsequently driving the expression of oncogenic target genes. The resulting upregulation of CTGF and CYR61 likely contributes to therapeutic resistance through extracellular matrix remodeling and pro-survival signaling, potentially explaining the aggressive behavior of residual tumors following incomplete ablation. This mechanistic insight provides a molecular framework for understanding post-iRFA tumor recurrence and highlights YAP as a potential therapeutic target in combination with thermal ablation therapies.

### iRFA accelerated the progression of HCC residual tumor by promoting the accumulation of MDSCs

**Figure 3:**
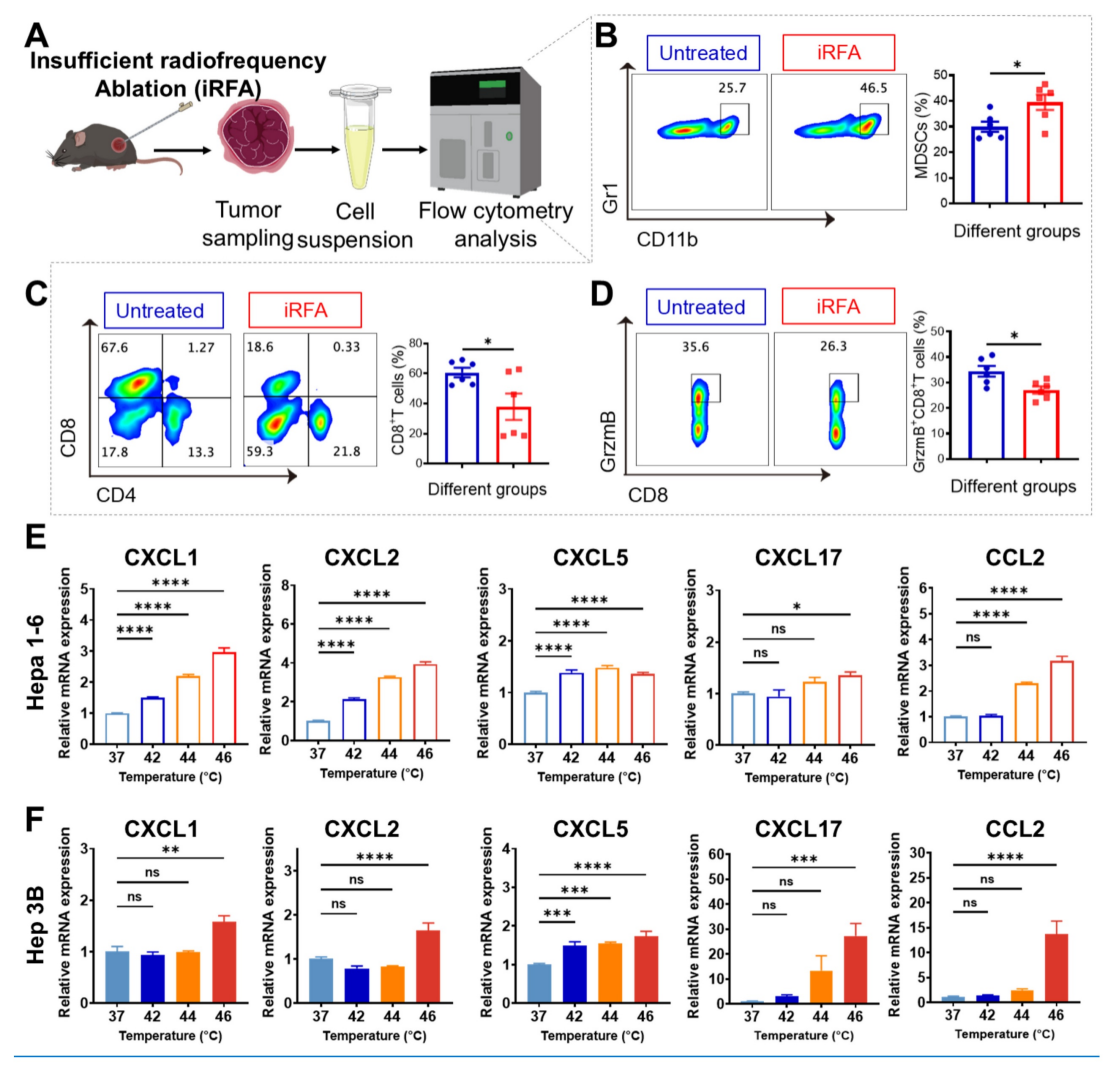
** iRFA promotes infiltration of immunosuppressive cells (MDSCs) in residual tumors.** (A) Schematic diagram of *in vivo* experimental workflow for flow cytometry analysis. (B) Flow cytometric analysis and quantification of MDSCs (CD11b⁺Gr1⁺) 7 days post-iRFA. (C) Flow cytometric assessment of CD8⁺ T-cell frequency 7 days after iRFA. (D) Flow cytometry plots and statistical data showing GrzmB⁺CD8⁺ T cells at day 7 post-iRFA (n = 6). (E) qPCR analysis of MDSC-associated chemokine expression in thermally treated Hepa1-6 cells. (F) qPCR analysis of MDSC-associated chemokine expression in thermally treated Hep3B cells (n = 3). Statistical significance: ns, not significant; *P < 0.05; **P < 0.01; ***P < 0.001; ****P < 0.0001.

Our previous work established that rapid progression of residual tumors following iRFA is associated with an immunosuppressive microenvironment, though the mechanistic drivers remain incompletely understood 40,45. Building on our findings of YAP activation in post-iRFA residual tumors (Figure 2B), we explored the potential link between YAP signaling and immune evasion. Moreover, analysis using the TIMER database indicated a strong positive correlation of YAP1 levels with MDSC infiltration (*P* < 0.001) (Figure S3), providing computational evidence linking YAP signaling to immunosuppression. Given that elevated MDSCs levels are clinically linked to poorer recurrence-free survival in HCC patients following RFA treatment 46, we characterized the immune landscape of residual tumors following iRFA. A subcutaneous HCC model was established, after which iRFA treatment was performed. Tumor tissues were harvested 7 days following treatment in order to conduct an immune profile via flow cytometry (Figure 3A). The strategy employed for the identification of key immune populations is presented in more detail in Figure S4. Compared to untreated controls, iRFA-treated tumors exhibited a marked increase in the proportion of MDSCs (CD11b^+^Gr1^+^) compared to untreated controls (Figure 3B). We further observed that iRFA predominantly induced significant infiltration of PMN-MDSCs, whereas M-MDSCs exhibited only a modest and statistically non-significant increase (Figure S5). This expansion of MDSCs was accompanied by a marked reduction in cytotoxic CD8^+^ T cells (CD3^+^CD8^+^) (Figure 3C) and diminished granzyme B expression in CTLs, indicative of impaired antitumor immunity (Figure 3D). In contrast, the proportions of DC cells (CD45⁺CD11c⁺) and macrophages (CD11b⁺F4/80⁺) within tumors remained comparable among the iRFA-treated groups (Figure S6 A-B), highlighting the selective enrichment of MDSCs following iRFA. Together, these findings suggest that iRFA remodels the tumor immune landscape by fostering an MDSC-dominated immunosuppressive niche which may facilitate residual tumor escape and progression.

Given the observed correlation between YAP activation and MDSC infiltration, we hypothesized thermal stress triggers YAP-dependent chemokine secretion to recruit MDSCs. To test this, we subjected Hepa1-6 and Hep3B HCC cell lines to sublethal heat treatment *in vitro* and assessed the expression of MDSC-associated chemokines. Both cell lines exhibited significant upregulation of CXCL1, CXCL2, CXCL5, CXCL17, and CCL2 following thermal stimulation (Figure 3E-F), consistent with prior reports linking these chemokines to MDSC trafficking 47-52. Quantitative analysis revealed a significant upregulation of all five chemokines in both cell lines upon thermal stimulation at 46 °C, with CXCL5 increased 1.4-fold in Hepa1-6 (*P* < 0.001) and 1.7-fold in Hep3B (*P* < 0.001), suggesting a direct link between heat stress and MDSC-attracting chemokine production. These findings further support the hypothesis that iRFA recruits MDSCs by upregulating MDSC-related chemokines, thereby fostering an immunosuppressive milieu that sustains the persistence and proliferation of residual tumor cells.

### YAP inhibition attenuates the tumor immunosuppressive microenvironment via suppression of MDSCs recruitment and infiltration

**Figure 4:**
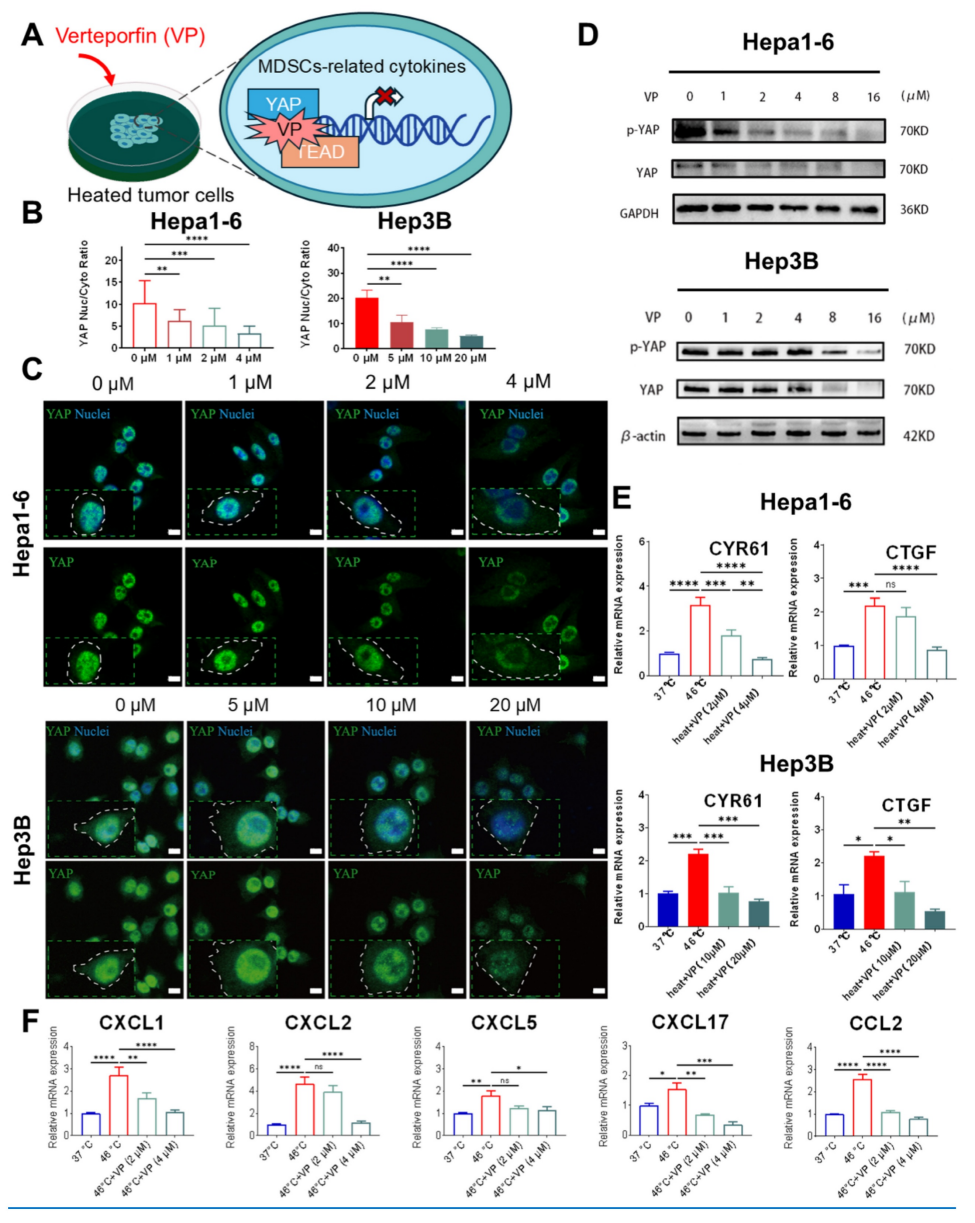
** YAP-mediated MDSCs infiltration in residual tumor post-ablation.** (A) Illustration of the experimental procedure for evaluation the effects of VP on YAP and MDSC-related chemokines expression *in vitro*. (C) Representative images of immunostained YAP in heated Hepa1-6 and Hep 3B cells and their quantification of the nucleo-cytoplasmic ratio (B) following VP treatment. Scale bar,10 μm. (D) relative protein content of pYAP and YAP in Hepa1-6 and Hep3B cells treated with VP. (E) qPCR analysis showing relative expression CYR61 and CTGF following VP treatment in both heated cells. (F) Quantitative PCR results illustrating the mRNA levels of MDSC-associated chemokines following VP treatment in heated Hepa1-6 cells (n = 3). Statistical significance: ns: not significant. *P < 0.05. **P < 0.01. ***P < 0.001. ****P < 0.0001.

Having observed concomitant YAP activation and increased MDSCs infiltration in residual tumor following iRFA, our subsequent research investigated the potential mechanistic between these phenomena. Initially, we evaluated the effects of VP on YAP activity in both murine-derived Hepa1-6 and human-originated Hep3B cells post-heat treatment (Figure 4A). IF analysis revealed a marked decrease in YAP nuclear localization upon VP treatment in both cell lines, indicating effective suppression of YAP activation (Figure 4B-C). WB analysis further demonstrated a reduction in YAP protein levels and a decrease in p-YAP in both cell lines (Figure 4D). Subsequently, RT-qPCR analysis of canonical YAP transcriptional targets showed downregulation of CYR61 and CTGF expression in VP-treated cells (Figure 4E), further confirming functional YAP suppression. To further clarify how YAP regulates the recruitment of MDSCs, we then analyzed the mRNA expression levels of previously identified MDSC-associated chemokines (CXCL1, CXCL2, CXCL5, CXCL17, and CCL2) following VP treatment using RT-qPCR. Consistent with our hypothesis, VP treatment induced significantly downregulated the expression of these chemokines in both Hepa1-6 and Hep3B cells (Figure 4F and Figure S7).

To further substantiate the mechanism, we generated three YAP-targeting shRNAs (YAP-sh1/2/3) and validated knockdown efficiency by qPCR. YAP-sh2 achieved the highest knockdown, approximately 79%. Upon YAP depletion, the transcript levels of MDSC-related chemokines were reduced, mirroring the pattern observed after VP treatment (Figure S8). To further support our conclusions, we performed qPCR analysis of Arg1 and iNOS expression in MDSCs after *in vitro* co-culture with different Hepa1-6 conditions (Figure S9). Compared with MDSCs co-cultured with untreated Hepa1-6 cells, Arg1 and iNOS expression were markedly upregulated when co-cultured with heat-treated Hepa1-6 cells, whereas co-culture with heat-treated YAP-knockdown (heat-shYAP) Hepa1-6 cells significantly reduced their expression. Collectively, the results indicate that heat stimulation enhances the immunosuppressive activation of MDSCs, while YAP inhibition attenuates their suppressive function.

### Preparation and characterization of QCS/TA@VP hydrogel

Verteporfin (VP), a hydrophobic compound with poor aqueous solubility, faces dual challenges of ineffective target-site bioavailability and rapid clearance via intravenous injection. The QCS/TA hydrogel formulation significantly improves VP solubility and enables localized sustained drug release, thereby overcoming these limitations while reducing systemic exposure; SEM images (Figure S10A-B) showed a loose and highly porous network, providing abundant free volume and interconnectivity that favor drug loading and sustained release. Furthermore, the post-ablation cavity created by iRFA provides an anatomical reservoir for hydrogel-based drug delivery. The QCS/TA hydrogel can be administered through the original iRFA access route, conformally filling the irregular cavity geometry due to its moldable viscoelasticity. This spatiotemporal co-localization of therapeutic agents with residual tumor margins not only ensures sustained drug exposure at the tumor site but also positions the hydrogel as a promising synergistic adjuvant to iRFA therapy.

**Figure 5:**
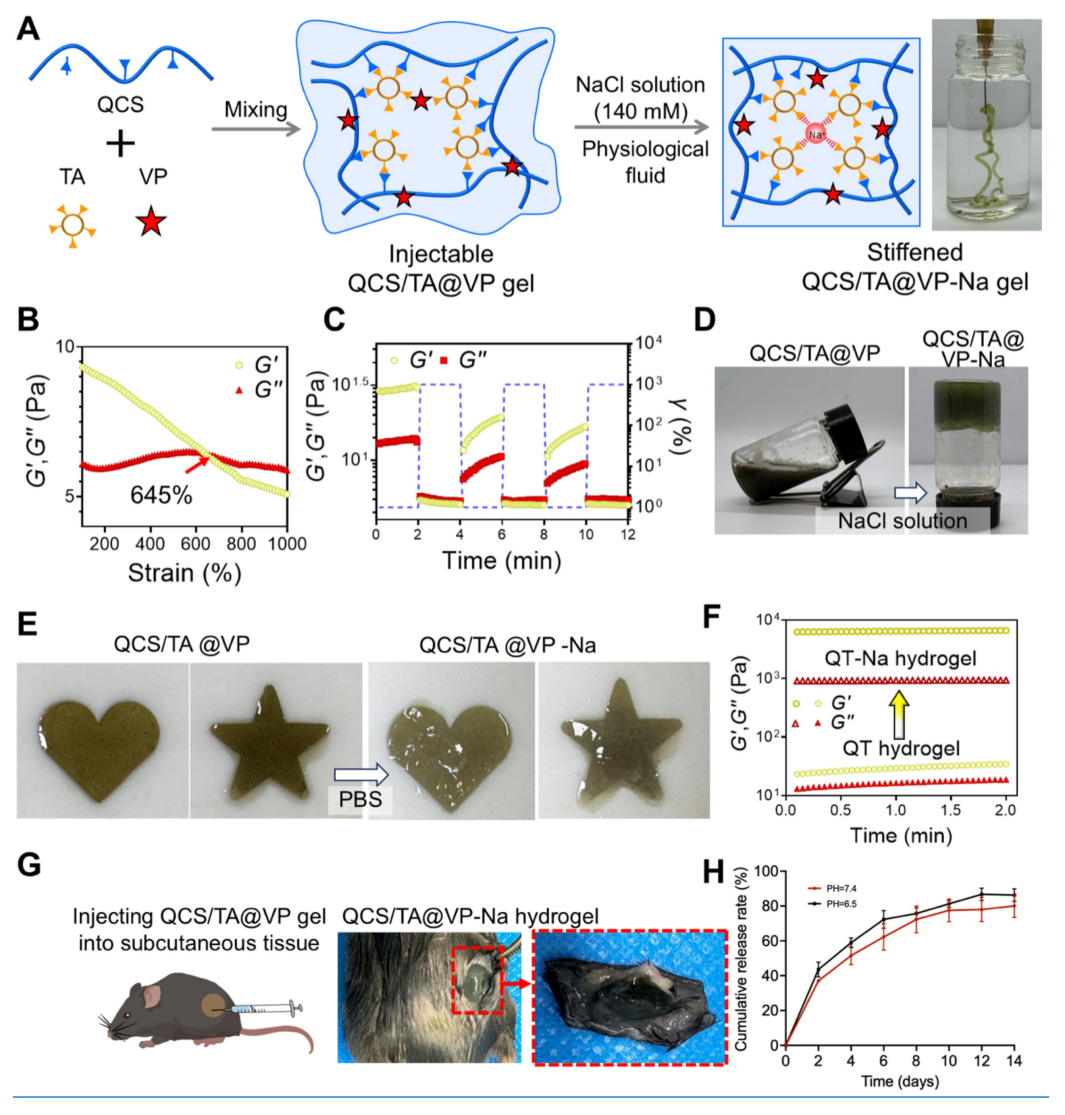
** Preparation and characterization of QCS/TA@VP and its derived QCS/TA@VP-Na hydrogel.** (A) Schematic of the microstructure of the QCS/TA@VP and its derived QCS/TA@VP-Na hydrogel. (B) Train-sweep measurements of the QCS/TA hydrogel (frequency = 1 Hz). (C) Repeated dynamic strain step testing (*γ* = 1% or 1000%, frequency = 1 Hz) of the QCS/TA@VP hydrogel. (D) Transformation from QCS/TA@VP hydrogel to QCS/TA@VP-Na hydrogel upon contacting NaCl aqueous solution (140 mM). (E) Injection of the QCS/TA@VP hydrogel into molds for casting QCS/TA@VP-Na hydrogels with various shapes. (F) G′ and G′′ of the QT and QT-Na hydrogels. (G) Transformation from QCS/TA@VP hydrogel to QCS/TA@VP-Na hydrogel *in vivo*. (H) *In vitro* cumulative release profiles of verteporfin (VP) from QCS/TA@VP hydrogel in 140 mM NaCl buffers adjusted to pH 7.4 and pH 6.5 (37 °C; n = 3).

According to our previous work, by simple mixing QCS, TA and VP aqueous solution, an injectable QCS/TA@VP hydrogel was prepared, owing to their moderate electrostatic interactions and hydrogen bonds 53. FTIR spectra of the drug-free QCS/TA hydrogel (Figure S10C) showed that the QCS bands at ~1484 cm⁻¹ (quaternary ammonium) and ~1649 cm⁻¹ (amide I/N-H bending region) were markedly attenuated and broadened/partly overlapped relative to neat QCS, indicating strong ionic complexation and hydrogen bonding with TA. Meanwhile, the O-H/N-H stretching appeared as a broader envelope centered at ~3382 cm⁻¹ (vs. 3565 and 3350 cm⁻¹ for QCS and TA), and no new characteristic bands were observed, supporting physical complexation rather than covalent bond formation. Due to relatively weak interactions, the QCS/TA@VP hydrogel can be injected through a 30 G syringe needle (right in Figure 5A). Moreover, as illustrated in the rheological strain sweep curve, the storage modulus (*Gʹ*) and loss modulus (*Gʹʹ*) of the QCS/TA@VP hydrogel intersected at approximately 645% strain (γ) (Figure 5B). Additionally, the cyclic strain tests revealed that QCS/TA@VP hydrogel exhibited a rapid and reversible transition between solid-like (Gʹ > Gʹʹ) and liquid-like (Gʹʹ > Gʹ) states. Upon applying a high strain (γ = 1000%), the network structure was disrupted, showing liquid-like behavior, whereas the storage modulus recovered once the strain was reduced to 1%, indicating the restoration of its solid-like characteristics (Figure 5C). These results showed the shear-thinning and good injectable properties of the QCS/TA@VP hydrogel.

Moreover, upon contacting physiological fluid, such as NaCl aqueous solution with the concentration of 140 mM, the injectable and soft QCS/TA@VP can transform into a stiffened QCS/TA@VP-Na hydrogel. This transformation was that Na^+^ in the physiological fluid can form coordination bonds with the TA in QCS/TA@VP to increase its cross-linking densities, resulting in a phase transformation (left in Figure 5A). As shown in Figure 5D, the QCS/TA@VP hydrogel in the glass bottle presented a flowing state, and then changed to a stiffened state upon contacting NaCl aqueous solution (140 mM). Moreover, depositing the QCS/TA@VP hydrogel into molds and immersing the them into NaCl aqueous solution, corresponding heart-shaped and pentagram-shaped QCS/TA@VP-Na hydrogel can be obtained, indicating suggesting that the QCS/TA@VP hydrogel can fit complex target site (Figure 5E). Besides, the mechanical properties of the hydrogel increased upon contacting NaCl aqueous solution (Figure 5F). For example, the *Gʹ* increased from 31.08±5.49 to 6432.41±109.37 Pa. In addition, we directly injected QCS/TA@VP hydrogel into the subcutaneous tissue of the mouse. Three minutes later, the skin of mice was cut open, and there was a complete and stiffened QCS/TA@VP hydrogel inside (Figure 5G).

The compression tests showed that in the 5-10% strain linear region, the hydrogel exhibited a small-strain compressive modulus of 7.97 kPa (R²=0.946; Figure S10D). Using σ=Eε, the estimated stresses at 1%, 2%, and 5% strain are ~0.08, 0.16, and 0.40 kPa, respectively—within the linear elastic regime and consistent with the small-amplitude cyclic loading (~1-5% strain) used in our model. These results suggested a low risk of instability or displacement under liver-like dynamic compression, supporting local depot retention. Under tension within 0-10% engineering strain, the hydrogel showed a Young's modulus of 170.83 kPa (R²=0.958), an ultimate tensile strength of 96.96 kPa, and an elongation at break of 107.33% (Figure S10E), indicating stable small-deformation mechanics and adequate tensile robustness for *in vivo* depot retention and dosing stability.

Finally, we evaluated the ability of the hydrogel to release the VP *in vitro*. After being immersed in NaCl solution, 80% of the VP was released only by the ninth day, which demonstrates its excellent sustained-release performance (Figure 5H). To mimic the mildly acidic tumor microenvironment, we additionally tested release at pH 6.5. The release profile at pH 6.5 showed a modest acceleration relative to that at pH 7.4, yielding a slightly higher cumulative fraction at all matched time points (Figure 5H). To further characterize the *in vivo* behavior of the hydrogel system, we performed optical *in vivo* fluorescence imaging to monitor drug retention and release kinetics. Indocyanine green (ICG) was used as a fluorescent tracer to mimic VP. As shown in Figure S11, the fluorescence signal at the injection site rapidly diminished in the free ICG group, indicating rapid drug diffusion. In contrast, the ICG@Gel group maintained a strong fluorescence signal within the tumor region for up to 14 days, demonstrating the hydrogel's ability to prolong local drug retention and enable sustained *in vivo* release. These results confirm that the QCS/TA-based hydrogel provides a favorable in *vivo* delivery profile with extended retention and controlled release capacity. Together, these data indicated that QCS/TA@VP provided robust sustained delivery across neutral and mildly acidic environments, supporting it use as a local drug-delivery carrier to prolong drug residence time, thereby potentially helping to reduce tumor recurrence.

We evaluated the degradation behavior of QCS/TA hydrogels. When incubated in PBS at pH 7.4 and pH 6.5 (37 °C), the hydrogel gradually degraded over time (Figure S12A). Furthermore, the *in vivo* degradability of QCS/TA and QCS/TA@VP hydrogels was evaluated by injecting them into the subcutaneous tissue on the backs of mice (Figure S12B). In the macroscopic images, the volume of the QCS/TA and QCS/TA@VP hydrogel (red dotted circle) in the subcutaneous tissue gradually decreased from 7 to 56d (Figure S13). At day 56, the QCS/TA hydrogel showed 25.47 ± 4.60% degradation, and the QCS/TA@VP hydrogel showed 24.40 ± 4.31% degradation, with no significant difference between groups. Thus, hydrogels can undergo gradual biodegradation both *in vitro* and *in vivo*.

### QCS/TA@VP hydrogel activates anti-tumor immunity and suppresses residual tumors after iRFA

**Figure 6:**
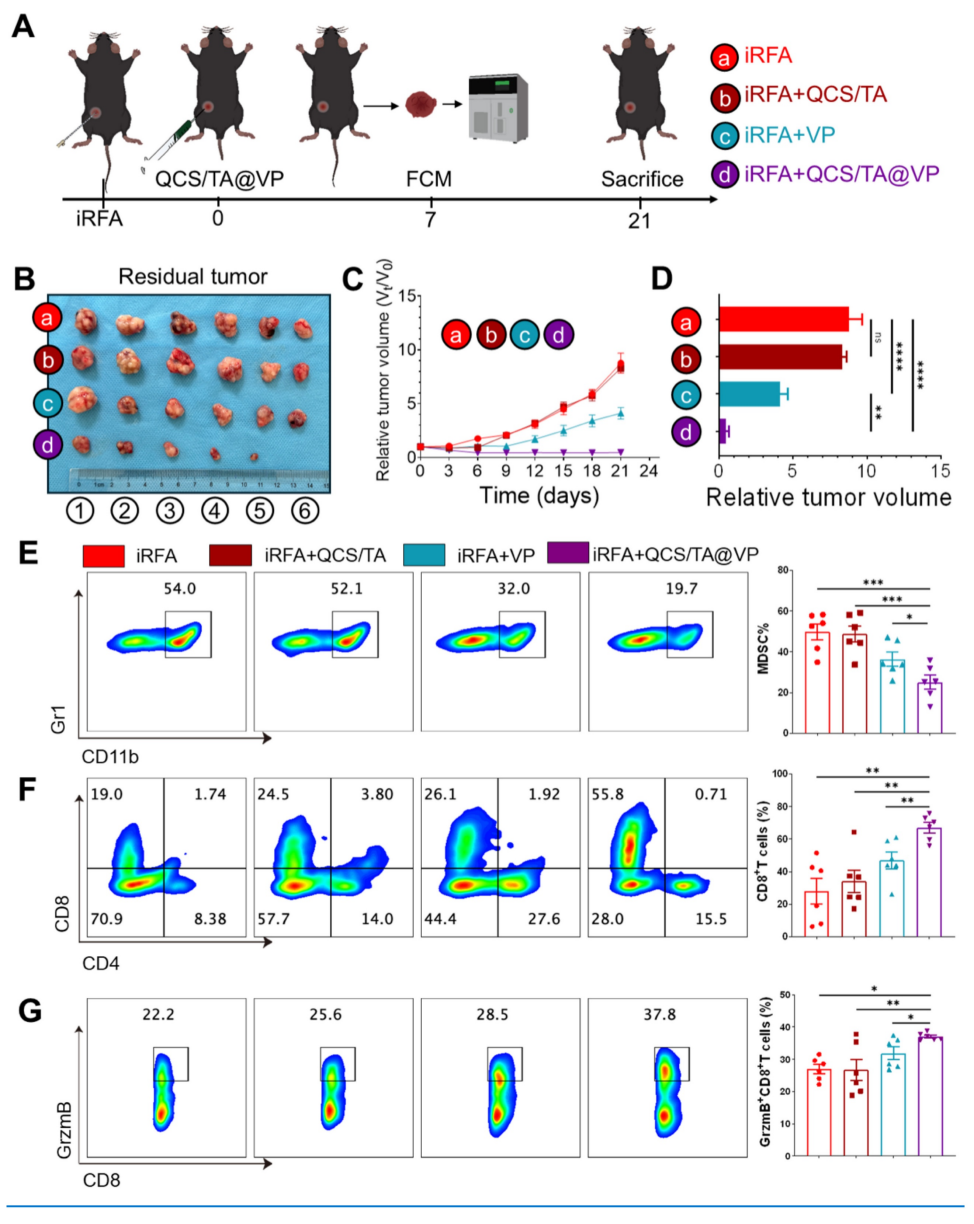
** QCS/TA@VP hydrogel activates anti-tumor immunity of residual tumors after iRFA.** (A) Schematic diagram of the animal experimental design. (B) Representative tumors images from each treatment group. (C) Growth curves of residual HCC tumors following various treatments. (D) Tumor volume statistics on day 21 post-treatment. (E) Representative flow cytometry and statistical plots illustrating MDSCs (CD11b^+^Gr1^+^) after different treatments. (F) Representative flow cytometry and statistical plots illustrating CD8^+^Tcells after different treatments. (G) Representative flow cytometry and statistical plots illustrating CTLs after different treatments (n = 6). Statistical significance: ns: not significant. *P < 0.05. **P < 0.01. ***P < 0.001. ****P < 0.0001.

To evaluate the therapeutic potential of QCS/TA@VP hydrogel as an adjuvant to iRFA, we conducted a preclinical therapeutic evaluation in a murine Hepa1-6 residual tumor model after iRFA using the standardized treatment protocol detailed in Figure 6A. Following iRFA treatment, PBS, QCS/TA, VP, and QCS/TA@VP were injected into residual tumors. Tumor growth curves indicated that compared to the PBS group, free VP monotherapy showed slight tumor growth inhibition but with no statistical difference; QCS/TA did not exhibit significant tumor suppression. Conversely, the residual tumor growth in the QCS/TA@VP group was markedly inhibited (Figure 6C). Terminal analysis of resected residual tumors at the experimental endpoint (Day 21) demonstrated that QCS/TA@VP treatment reduced residual tumor volumes by 95 ± 5% compared to PBS controls, QCS/TA vehicle, and free VP monotherapy groups, significantly outperforming both QCS/TA and free VP treatments (Figure 6B, D). Consistent with the aforementioned findings, Kaplan-Meier analysis showed that mice receiving QCS/TA@VP treatment had a markedly prolonged survival compared with the other groups. (Figure S14). Subsequently, we evaluated the anti-tumor immune effects post various treatments. As anticipated, the QCS/TA@VP group exhibited a significant reduction in MDSCs infiltration (27 % decrease vs. PBS controls, *P <* 0.001, Figure 6E) and a corresponding increase in CD8^+^ T cells (39 % increase vs. PBS controls, *P <* 0.01, Figure 6F) and CTLs (10% increase vs. PBS controls, *P <* 0.05, Figure 6G) following QCS/TA@VP treatment. To further validate the immunomodulatory effects of our hydrogel-based therapy, we additionally performed immunohistochemical (IHC) staining for Foxp3⁺ Tregs in tumor tissues. The results demonstrated that QCS/TA@VP treatment markedly reduced Treg infiltration compared with the other groups (Figure S15). Additionally, we collected these residual cancer tissues and conducted RT-qPCR analysis, which revealed a significant reduction in MDSCs-associated chemokines in the QCS/TA@VP treated group (Figure S16). These finding suggested that QCS/TA@VP not only alleviates MDSC-mediated suppression but also mitigates Treg-associated immunosuppression, further supporting its capacity to remodel the tumor immune microenvironment, suggesting that QCS/TA@VP hydrogel treatment can serve as a complementary therapeutic strategy to RFA, suppressing residual cancer growth post-iRFA through the activation of anti-tumor immunity.

Furthermore, the biocompatibility of the QCS/TA@VP drug delivery system was evaluated following subcutaneous injection in mice. Hematoxylin and eosin (H&E) staining and serum biochemical analyses of liver and kidney function revealed no significant difference between hydrogel-treated mice and blank controls across major organs (heart, liver, spleen, lung, kidney), with hepatic architectures remaining intact (Figure S17A, C). In contrast, mice receiving free VP (dissolved in 10% DMSO solvent) induced acute hepatotoxicity characterized by inflammatory infiltration and hepatocellular edema (Figure S17B).

In addition, we conducted an extended subcutaneous implantation study (up to 56 days) including both blank QCS/TA and drug-loaded QCS/TA@VP hydrogels. Serial serum biochemistry (liver and kidney panels) and H&E histology of major organs (heart, liver, spleen, lung, kidney) showed no significant abnormalities compared with blank controls throughout the observation window (Figure S18 and Figure S19). Histological analysis of the subcutaneous injection site showed that the QCS/TA hydrogel was well tolerated. H&E staining revealed intact tissue structure with minimal inflammatory cell infiltration, while Masson's trichrome staining showed uniform collagen distribution without significant fibrosis, indicating good local biocompatibility and safety of the hydrogel (Figure S20). This analysis indicates that hydrogel encapsulation effectively eliminates solvent-related toxicity and ensures favorable systemic biocompatibility.

## Discussion

This study elucidates the critical role of YAP in driving post-ablation tumor recurrence in HCC. Specifically, sublethal hyperthermia induced by iRFA activates YAP via dephosphorylation and subsequent nuclear translocation, conferring transcriptional co-activator activity. The activated YAP upregulates MDSCs-associated chemokines (e.g. CXCL1, CXCL2, CXCL5, CXCL17, CCL2), which collectively mediate the recruitment of CD11b⁺Gr1⁺ MDSCs while impairing CD8⁺ T cell cytotoxic function. This mechanism aligns with the YAP-MDSC axis previously proposed by Wang et.al 24,50,54 in prostate cancer, pancreatic ductal adenocarcinoma and liver ischemia-reperfusion injury. However, our study provided the first evidence of YAP's pivotal role in reshaping the immunosuppressive microenvironment within post-ablation residual HCC models, thereby expanding the oncogenic landscape of YAP signaling in the context of thermal ablation. In addition, prior work by Xu *et al.* has demonstrated that YAP regulates the expression of CXCL1, CXCL5 through interaction with ETV4 55. Our study further expanded the regulatory scope of YAP, confirming that it synergistically recruits MDSCs through multiple chemokines, thereby enriching the molecular network underlying YAP-mediated immunosuppression. While additional studies have linked CXCL2-CXCR2 and CCL2-CCR2 signaling to MDSC trafficking across tumor types 52,56-58, we did not directly test the necessity of any single chemokine in this work (e.g., by neutralization or receptor knockout), which we acknowledged as a limitation and a priority for future investigation.

Verteporfin (VP), a clinically approved photosensitizer and inhibitor of YAP-TEAD interaction, was found to effectively suppress nuclear translocation of YAP *in vitro* and the expression of MDSC-related chemokines was found to be downregulated. *In vivo* experiments demonstrated that VP significantly reduced MDSC infiltration while restoring the numbers and cytotoxic function of CD8^+^ T cells. Unlike Golino *et al.*'s study 34 on cholangiocarcinoma, this work integrates VP's immunomodulatory effects with the specific post-iRFA microenvironment, providing a clearer understanding of how it suppresses residual tumor progression via the YAP-MDSCs axis.

While repeated intraperitoneal VP administration showed therapeutic efficacy in our model, this approach presents clinical limitations including poor patient compliance and systemic toxicity risks. To address these limitations, we developed a localized, hydrogel-based delivery platform to achieve sustained therapeutic drug concentrations at the tumor site while minimizing off-target effects, thereby optimizing both treatment convenience and safety. Our injectable hydrogel, composed of QCS and TA, was synthesized using simple preparation procedures and readily available materials 53,59. This platform exhibited several key advantages: (1) Shear-thinning properties enabling injection through a 30G needle and conformal filling of irregular residual cavities; (2) Na⁺-coordination-triggered phase transition upon contact with physiological fluids, forming a rigid structure to achieve localized VP sustained release and significantly prolong drug retention time; (3) Enhanced antitumor immunomodulatory activity mediated by YAP inhibition through optimized VP delivery efficiency, resulting in approximately 95% reduction in tumor volume (mean relative growth: 0.5 vs. 9.0 in controls, *P* < 0.001) in the QCS/TA@VP group, with complete tumor regression observed in 16.7% (1/6) of treated subjects.

Nevertheless, the current study has some limitations: (1) Although* in vivo* biodegradation kinetics were quantified over 56 days, the systemic biosafety of isolated degradation products was not specifically assessed (e.g., by intraperitoneal or intravenous administration of degradation eluates); therefore, long-term toxicological evaluation is warranted in future studies. (2) While adding a pH 6.5 (37 °C) release condition improves the physiological relevance of the in-vitro assay, the present setup did not include serum proteins or proteases; as these can alter release via protein adsorption and enzyme-mediated degradation, we regard the current in-vitro curves as an upper-bound estimate of in-vivo release. We will next assess release in protein-containing and protease-supplemented media to disentangle mechanisms and improve the comparability and predictive value of the *in*-*vitro* model. (3) Subcutaneous models, while convenient for tumor monitoring, controlled sizing, intratumoral gelation, and repeated sampling, do not fully recapitulate the clinical microenvironment of hepatocellular carcinoma, particularly the hepatic vasculature, matrix composition, and perivascular heat-sink effects relevant to iRFA. Orthotopic intrahepatic models more closely mimic these conditions and are therefore essential for further validation. In this context, we will initiate studies using a rabbit orthotopic HCC/iRFA model to assess intrahepatic hydrogel distribution, retention, antitumor efficacy, and safety. These forthcoming experiments will provide additional support for the clinical translatability of our hydrogel-based therapeutic strategy.

Notably, unlike other hydrogels systems that require complex triggering conditions (e.g., external photothermal stimulation or enzymatic digestion), the QCS/TA@VP achieves controlled drug release under physiological conditions, demonstrating significant advantages for immediate intraoperative application and compatibility with combination RFA therapy.

## Conclusions

In conclusion, we identify the YAP-MDSC immunosuppressive axis as a major driver of post-iRFA tumor progression in HCC and establish YAP as a promising therapeutic target. Furthermore, by integrating oncogenic signaling modulation with biomaterial engineering, we introduce QCS/TA@VP hydrogel as a precision immunotherapy platform that synergizes with standard RFA procedures. This dual-modality approach—simultaneously targeting residual tumor cells and their immunosuppressive microenvironment—holds significant potential to redefine adjuvant strategies for incompletely ablated HCC.

## Supplementary Material

Supplementary figures and table.

## Abbreviations

HCC: hepatocellular carcinoma
RFA: radiofrequency ablation
iRFA: incomplete radiofrequency ablation
TAMs: tumor-associated macrophages
YAP: yes-associated protein
VP: verteporfin
QCS: quaternized chitosan
TA: tannic acid
IHC: immunohistochemical
IF: immunofluorescence
WB: western blot
