# Supplementary material for: Verteporfin-loaded hydrogel targeting YAP-mediated MDSCs recruitment for the treatment of residual tumors after incomplete radiofrequency ablation
Source: Theranostics. 2026 Jan 1;16(5):2372–87. doi: 10.7150/thno.119377 (PMC12712912; doi:10.7150/thno.119377)
Supplement: Supplementary file 1 — Supplementary figures and table. [file thnov16p2372s1.pdf]

Verteporfin-loaded hydrogel targeting YAP-mediated MDSCs recruitment for the treatment of residual tumors after incomplete radiofrequency ablation

Jiawen Chen[1,2,3,4,5, #], Junfeng Liu[6, #], Xiaoting Zhang[1,2,3,4, #], Xi Li[1,3,4], Jinming Fan[1,2,3,4], Shengchao Zhao[1,2,3,4], Junbin Liu[1,2,3,4], Bin Zhou[1,2,4*], and Ke Zhang[1,2,3,4*]

[1] Center of Interventional Medicine, The Fifth Affiliated Hospital of Sun Yat-sen University, Zhuhai, Guangdong Province, 519000, China

[2] Center of Cerebrovascular Disease, The Fifth Affiliated Hospital of Sun Yat-sen University, Zhuhai, Guangdong Province, 519000, China

[3] Guangdong Provincial Engineering Research Center of Molecular Imaging, The Fifth Affiliated Hospital of Sun Yat-sen University, Zhuhai, Guangdong Province, 519000, China

[4] Guangdong-Hong Kong-Macao University Joint Laboratory of Interventional Medicine, The Fifth Affiliated Hospital of Sun Yat-sen University, Zhuhai, Guangdong Province 519000, China

[5] Guangzhou University of Chinese Medicine-Shenzhen Hospital, Shenzhen, Guangdong Province, 518000, China

[6] Department of Interventional Therapy, Sichuan Clinical Research Center for Cancer, Sichuan Cancer Hospital & Institute, Sichuan Cancer Center, Affiliated Cancer Hospital of University of Electronic Science and Technology of China, Chengdu Province, 610041, China

[#] Jiawen Chen, Junfeng Liu and Xiaoting Zhang contributed equally to this work.

[*] Corresponding authors:

Bin Zhou, M.D., Ph.D.

Ke Zhang, M.D., Ph.D.

Center of Interventional Medicine, The Fifth Affiliated Hospital,

Sun Yat-sen University, Zhuhai, Guangdong Province, 519000, China

Email addresses for correspondence:

zhoub2@mail.sysu.edu.cn

zhangk276@mail.sysu.edu.cn

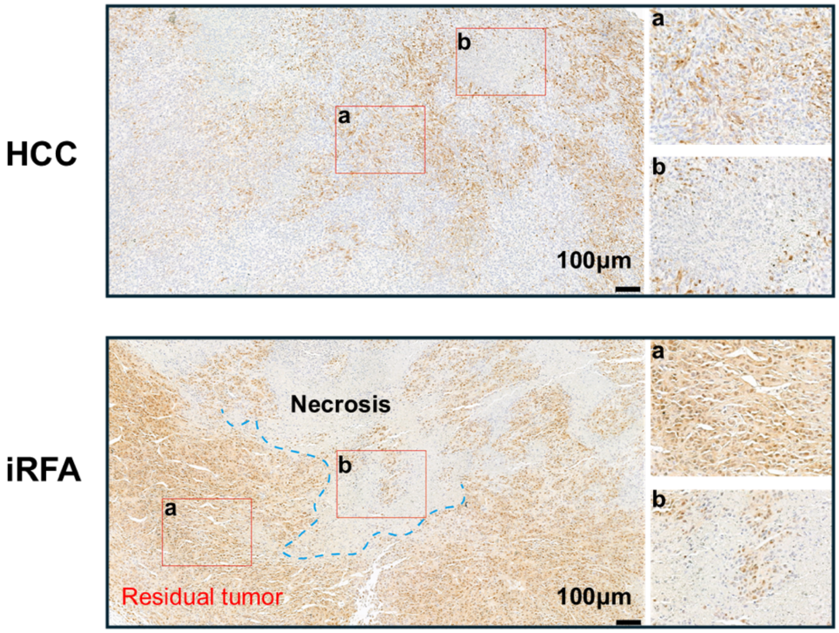

Figure. S1. Immunohistochemical assessment of tumor tissue after iRFA Immunohistochemical staining revealed disrupted tissue architecture, loss of cellular morphology, and pallor in staining in the iRFA group (b), compared to the untreated HCC group.

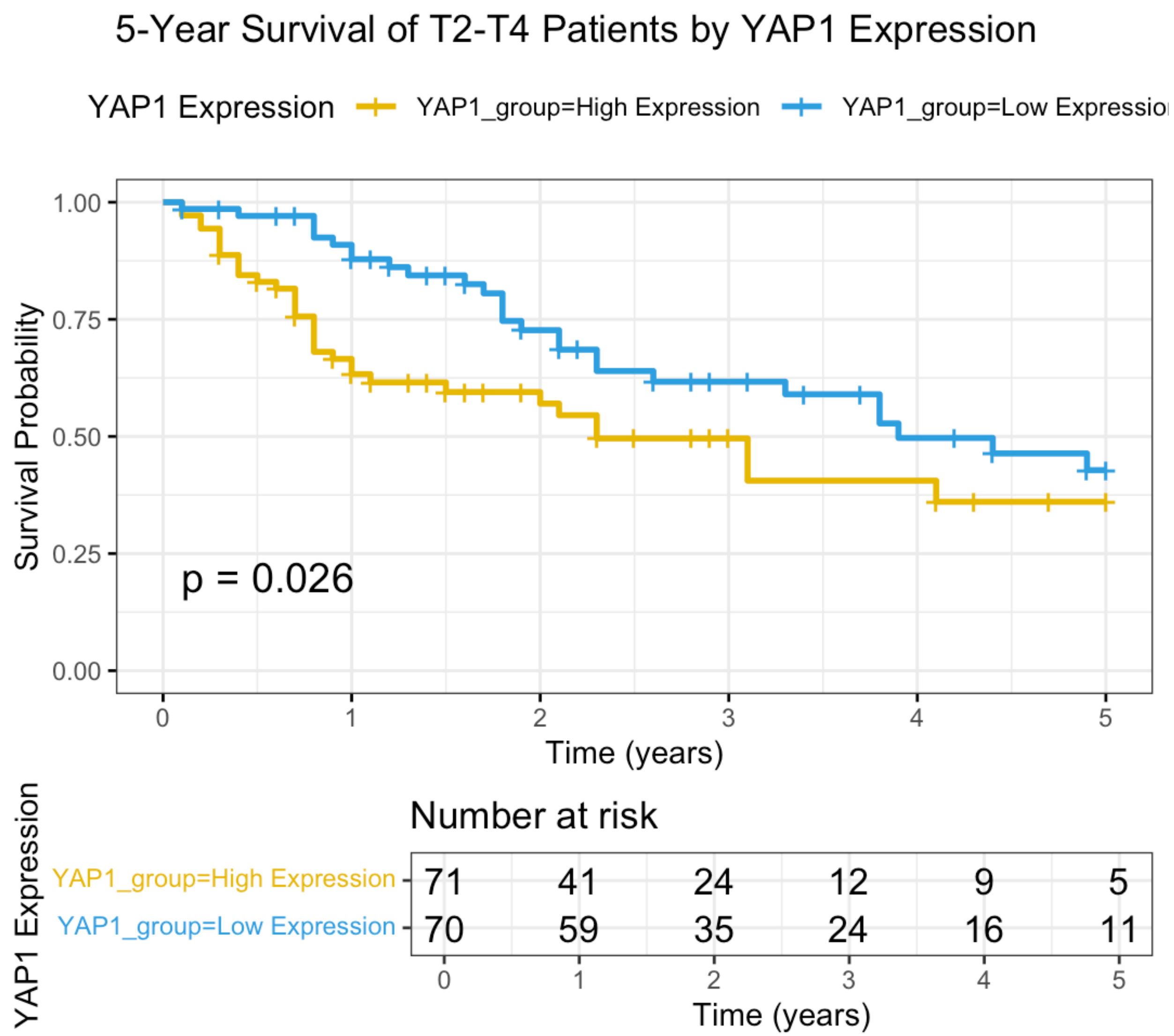

Figure. S2. Kaplan-Meier survival analysis of YAP expression in advanced HCC (T2-T4 stages).

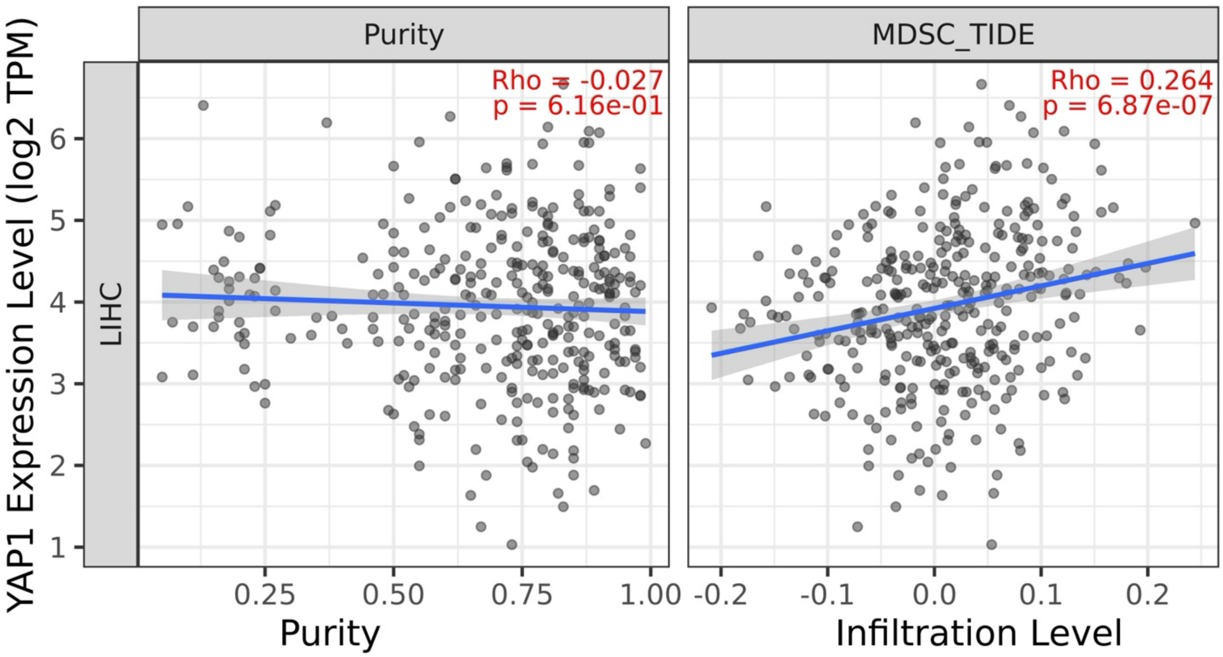

Figure. S3. Correlation between YAP1 expression with MDSCs infiltration. ***: p-value < 0.001.

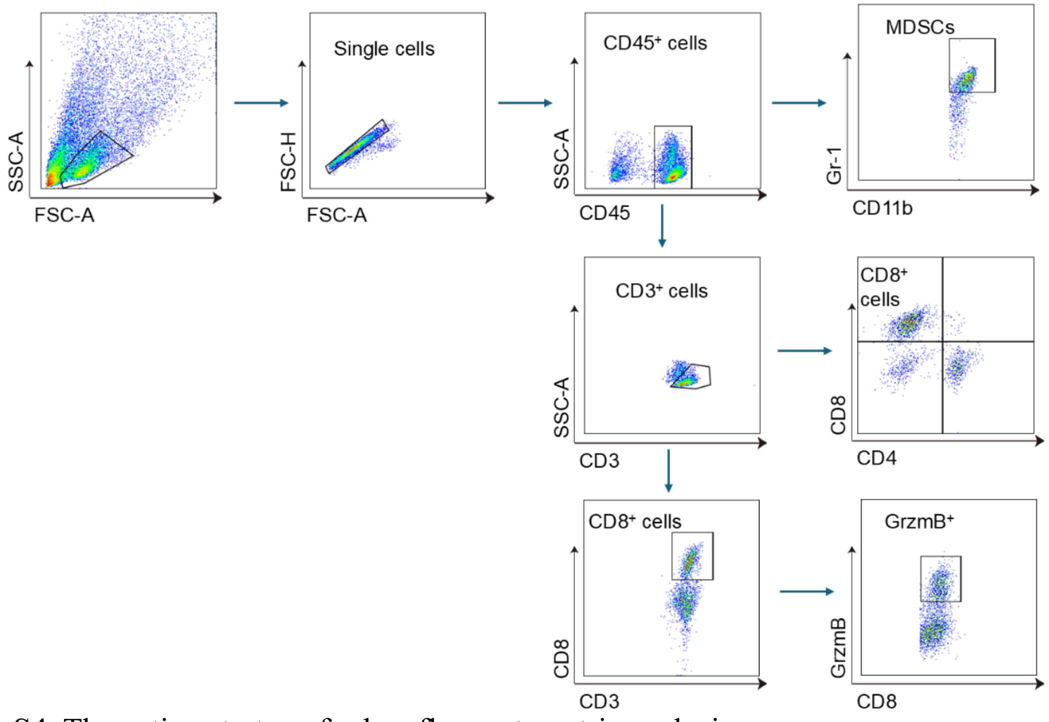

Figure. S4. The gating strategy for key flow cytometric analysis.

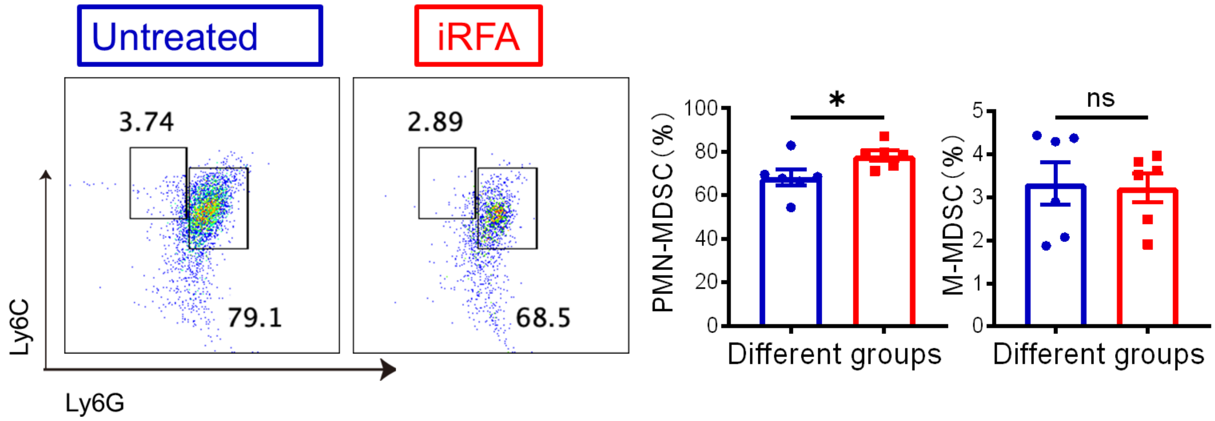

Figure. S5. Representative flow cytometry and Statistical plots illustrating MDSC subpopulation.

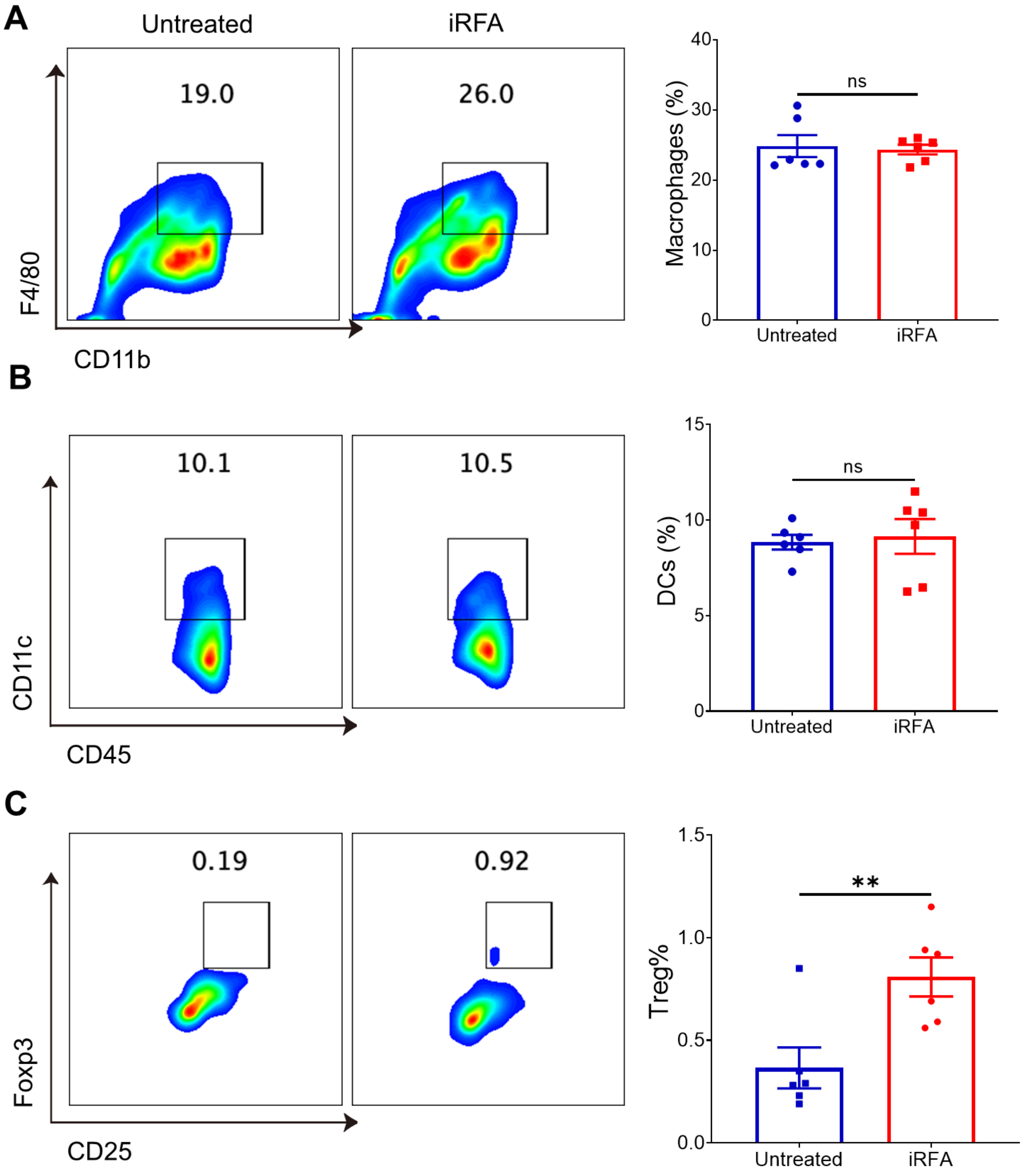

Figure. S6. (A) Representative flow cytometry and Statistical plots illustrating macrophages (F4/80^{+} CD11b^{+}) in residual tumors in untreated (Ctrl) and iRFA groups. (B) Representative flow cytometry and Statistical plots illustrating DCs (CD11c^{+} CD45^{+}) in residual tumors in untreated

(Ctrl) and iRFA groups. (C) Representative flow cytometry and Statistical plots illustrating Tregs ($Foxp3^+$ $CD25^+$) in residual tumors in untreated (Ctrl) and iRFA groups.

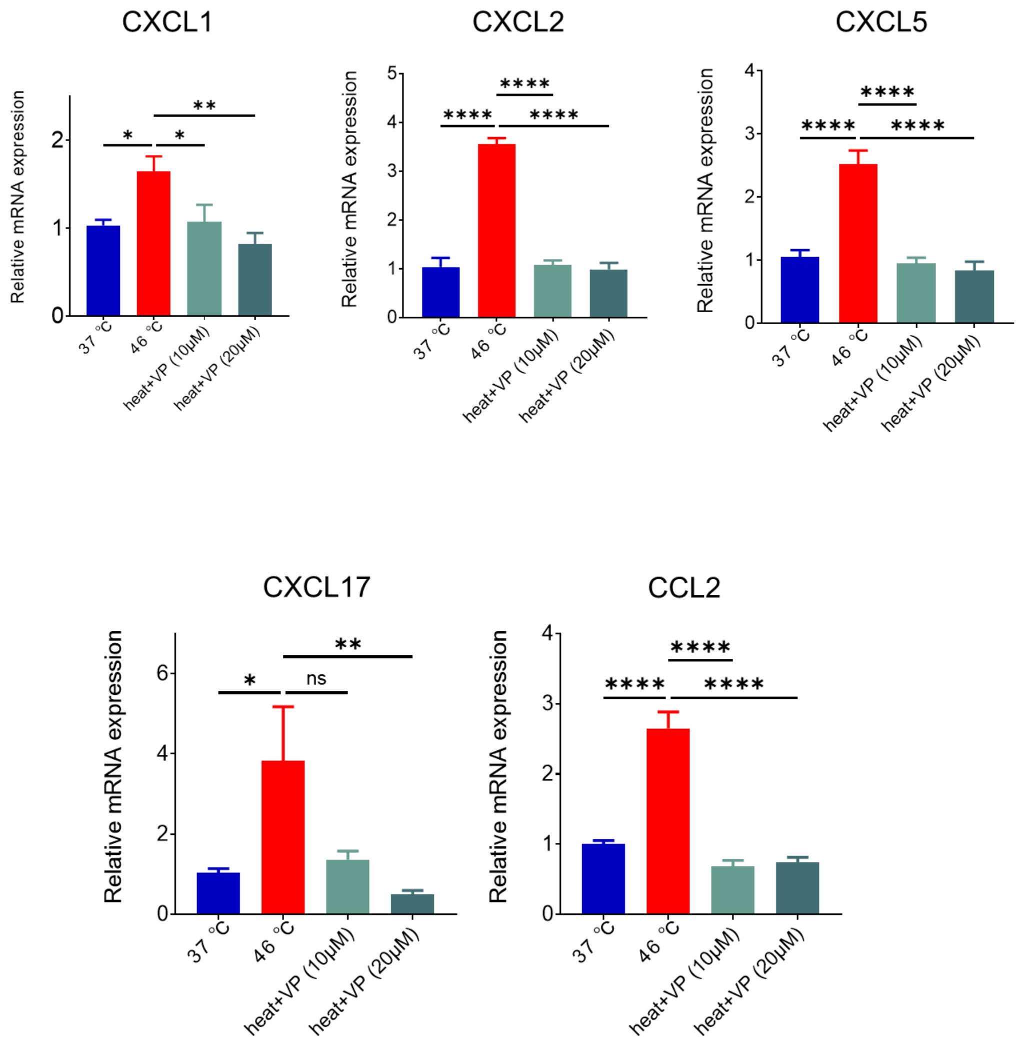
CXCL1
CXCL2
CXCL5
CXCL17
CCL2
Relative mRNA expression
37 °C
46 °C
heat+VP (10μM)
heat+VP (20μM)
ns

Figure. S7. qPCR analysis showing relative expression MDSCs-related cytokines following VP treatment in heated Hep3B cells. *P < 0.05, **P < 0.01, ***P < 0.001, ****P < 0.0001

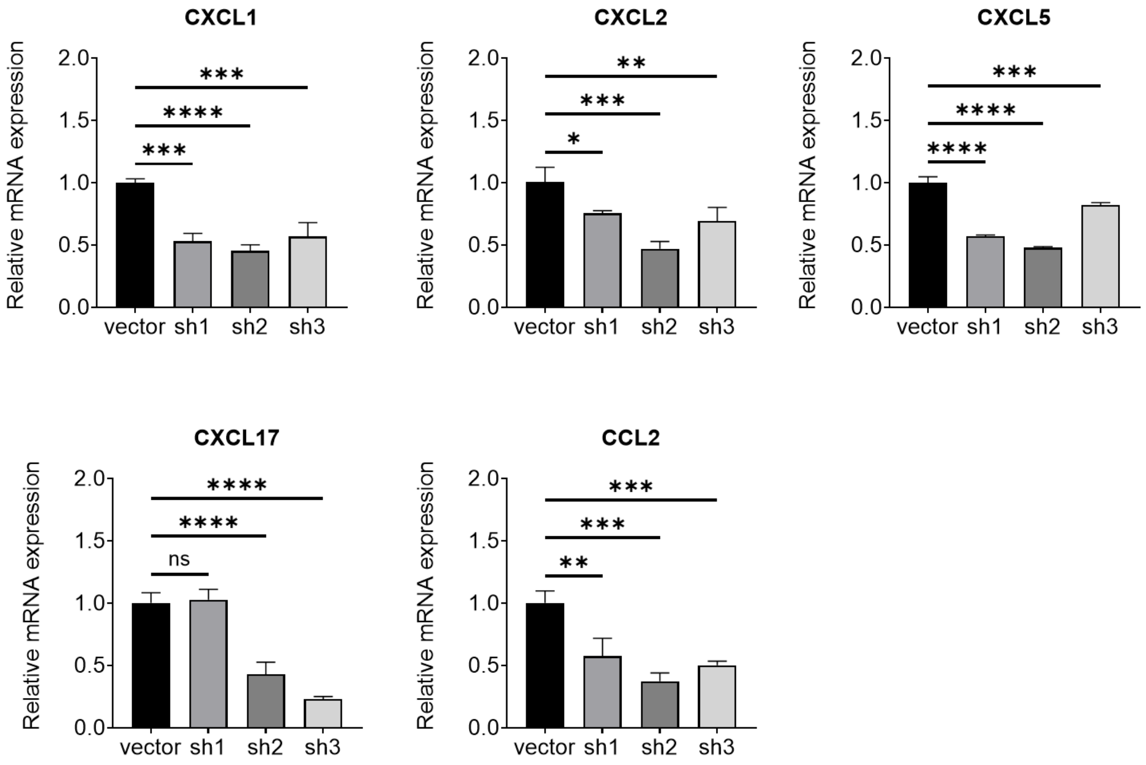

Figure. S8. qPCR analysis showing relative expression MDSCs-related cytokines following knockdown YAP

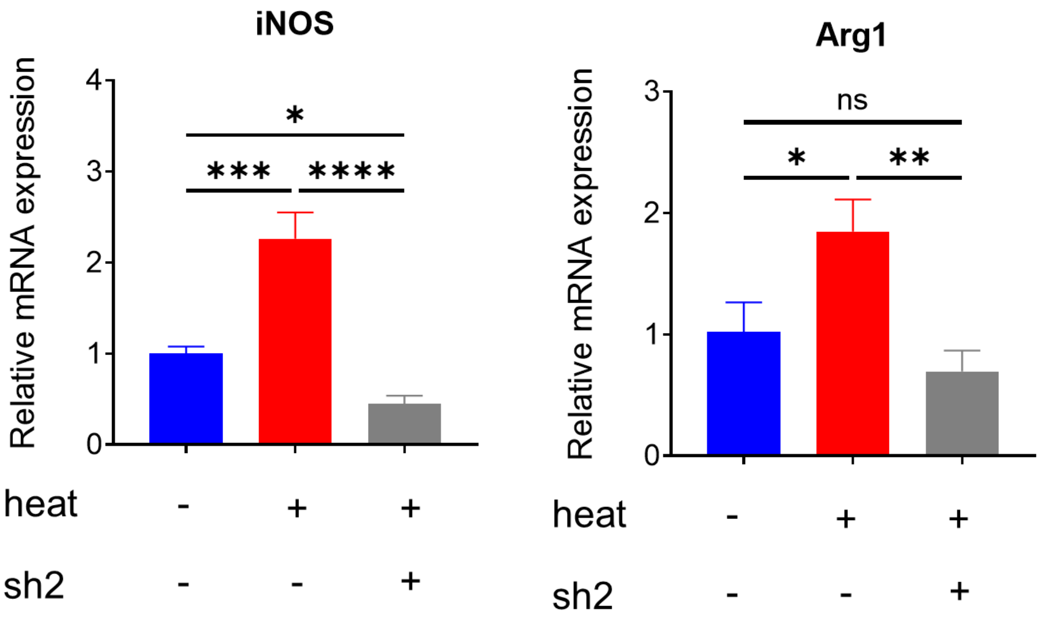

Figure. S9. qPCR analysis showing relative expression of iNOS and Arg1 following different treatment in MDSCs. *P < 0.05, **P < 0.01, ***P < 0.001, ****P < 0.0001

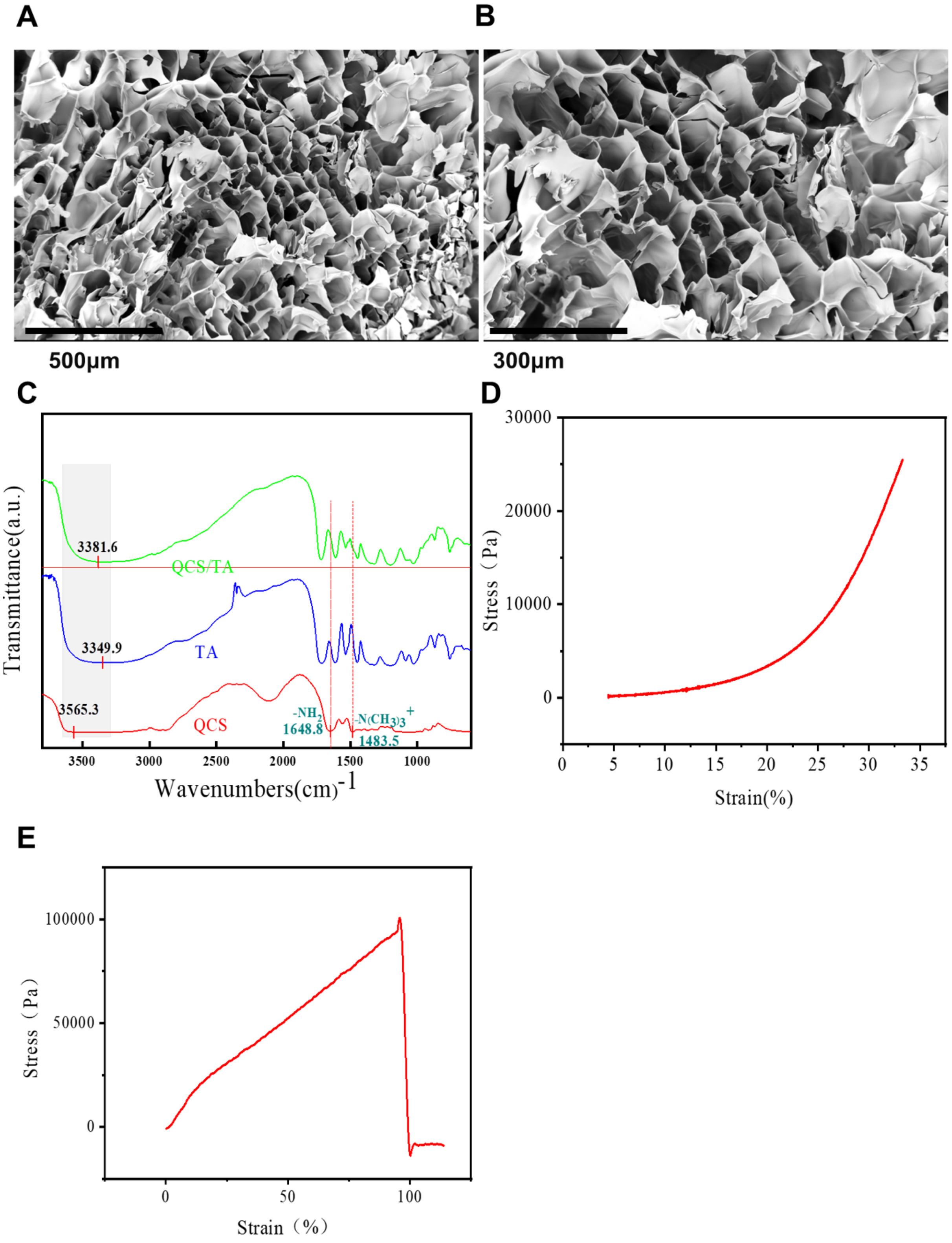
A
B
500μm
300μm
C
D
E
Transmittance(a.u.)
Wavenumbers(cm)-1
3381.6
QCS/TA
3349.9
TA
3565.3
QCS
-NH2
1648.8
-N(CH3)3+
1483.5
3500
3000
2500
2000
1500
1000
Stress（Pa）
Strain(%)
30000
20000
10000
0
5
10
15
20
25
30
35
100000
50000
0
50
100
Strain（%）

Figure. S10. The characterization of hydrogel. A.B The morphology of QCS/TA hydrogel was observed by SEM. (Scale bar: 500 μm (left) and 300 μm (right)). C. Fourier transform infrared (FTIR) spectra of the QCS, TA, and QCS/TA gel. D. Compression of QCS/TA hydrogels. E. Tensile testing of QCS/TA hydrogels

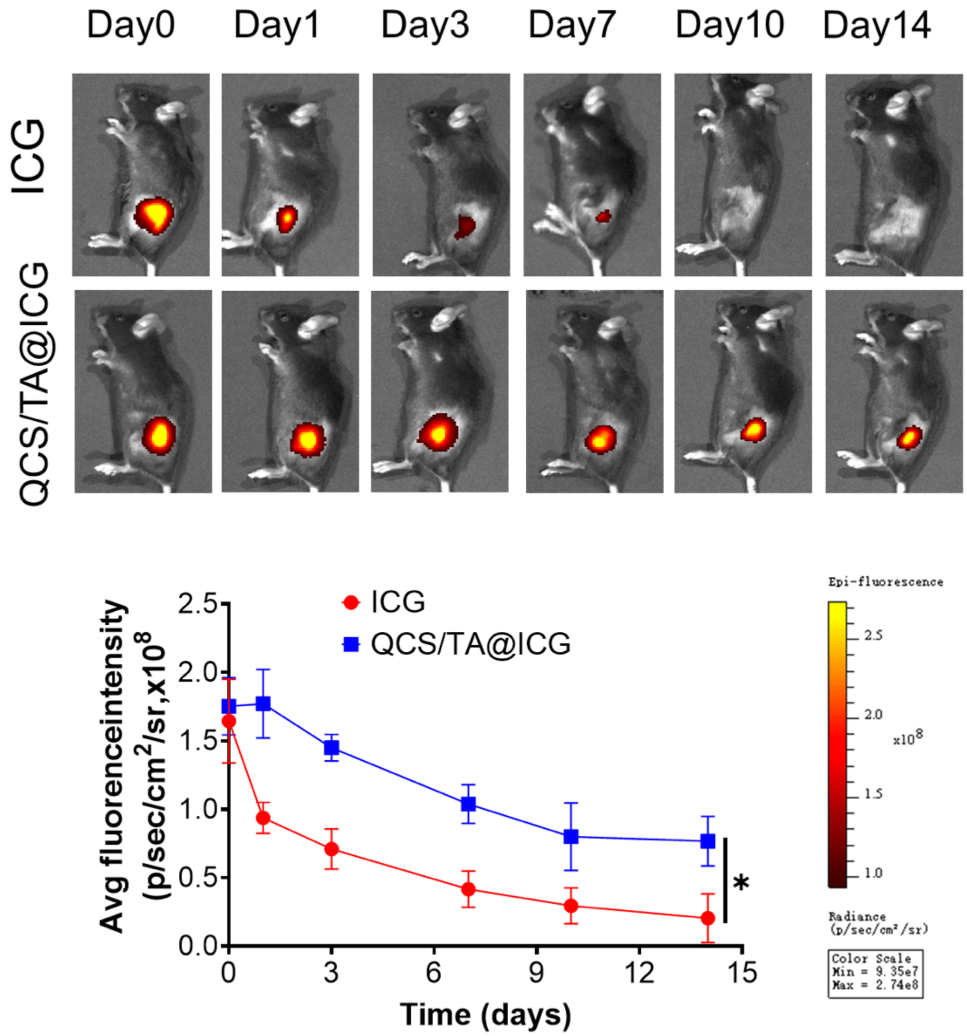

Figure. S11. IVIS images and statistical analysis of fluorescence signal recorded at different times after injection of ICG and ICG@Gel (n = 3).

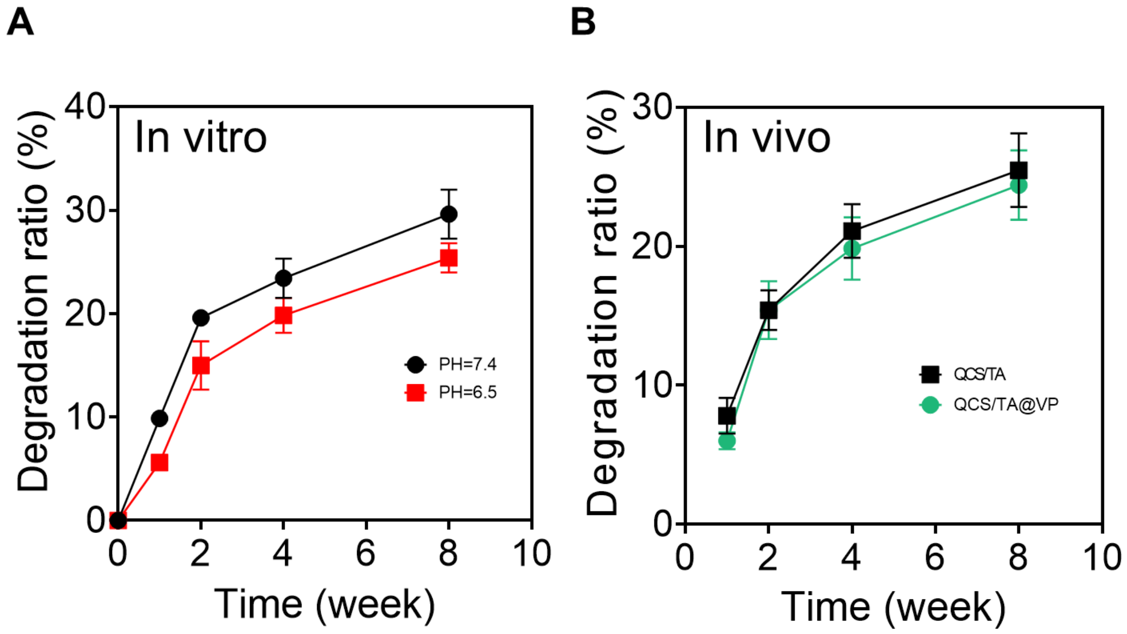

Figure. S12. Degradation of the QCS/TA and QCS/TA@VP hydrogel.

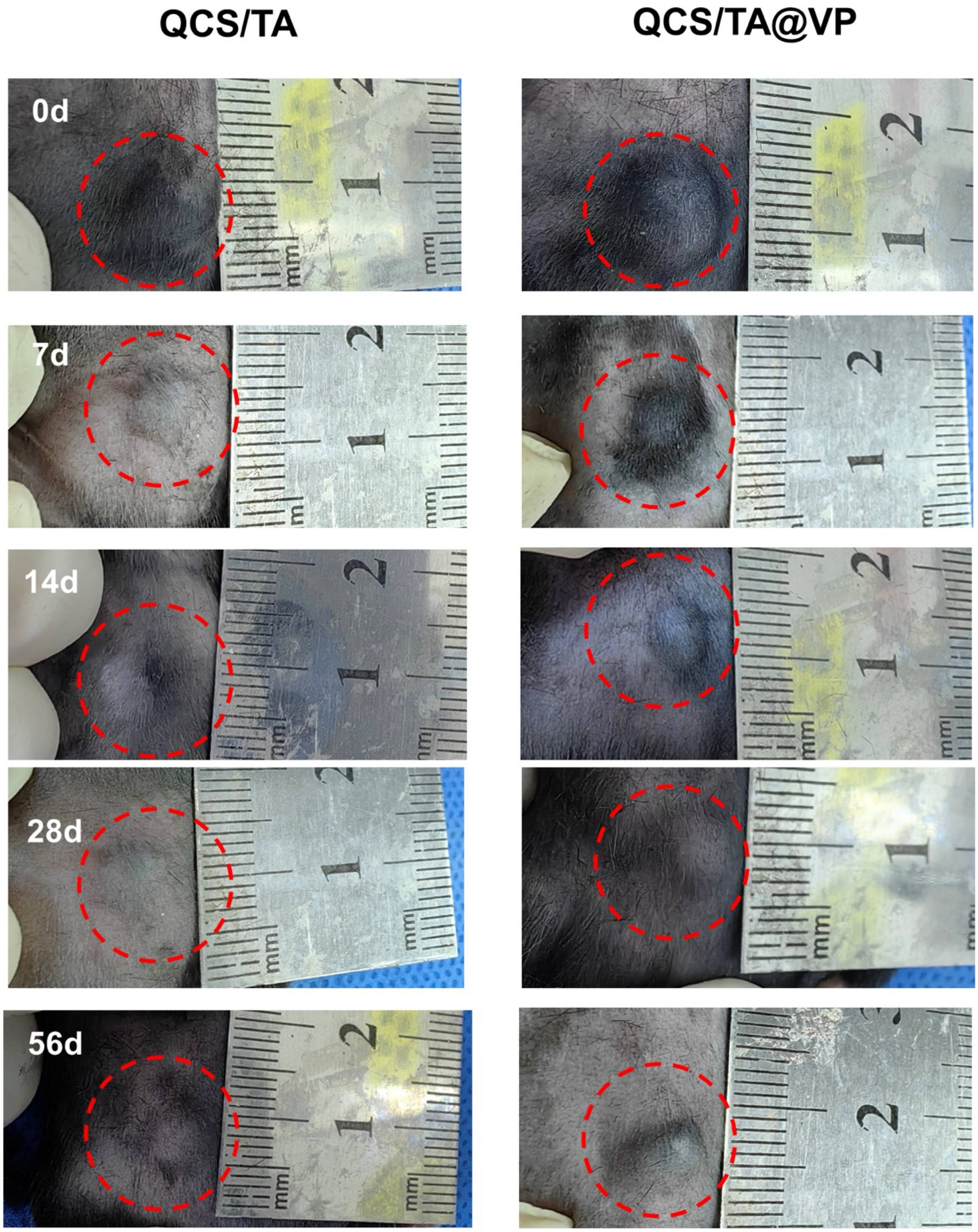

Figure. S13 Macroscopic images of the mice receiving QCS/TA and QCA/TA@VP hydrogel

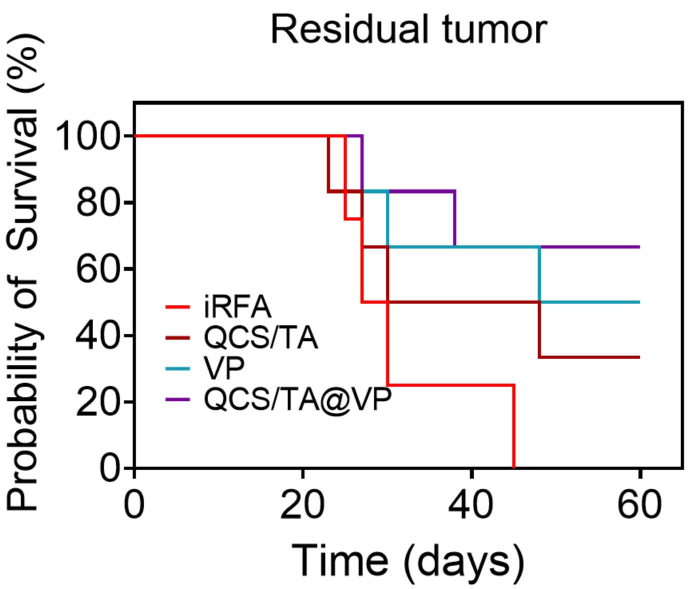

Figure. S14. Survival analysis of experimental mice in different groups (n = 6)

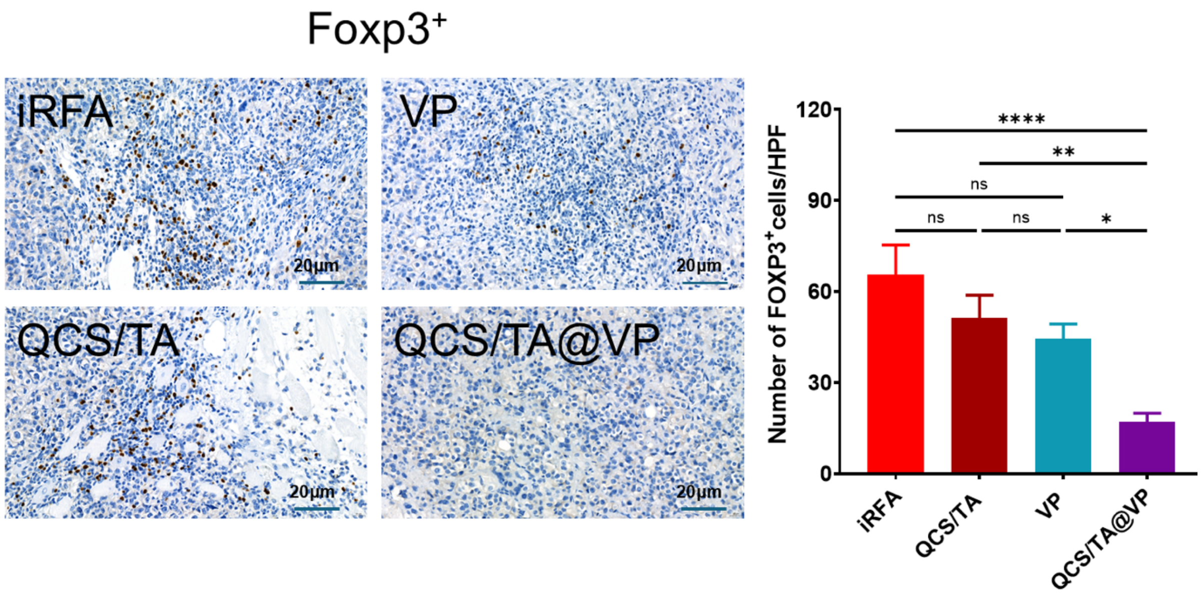

Figure. S15. IHC staining of Treg in residual tumor tissue, scale bar: 20 μm.

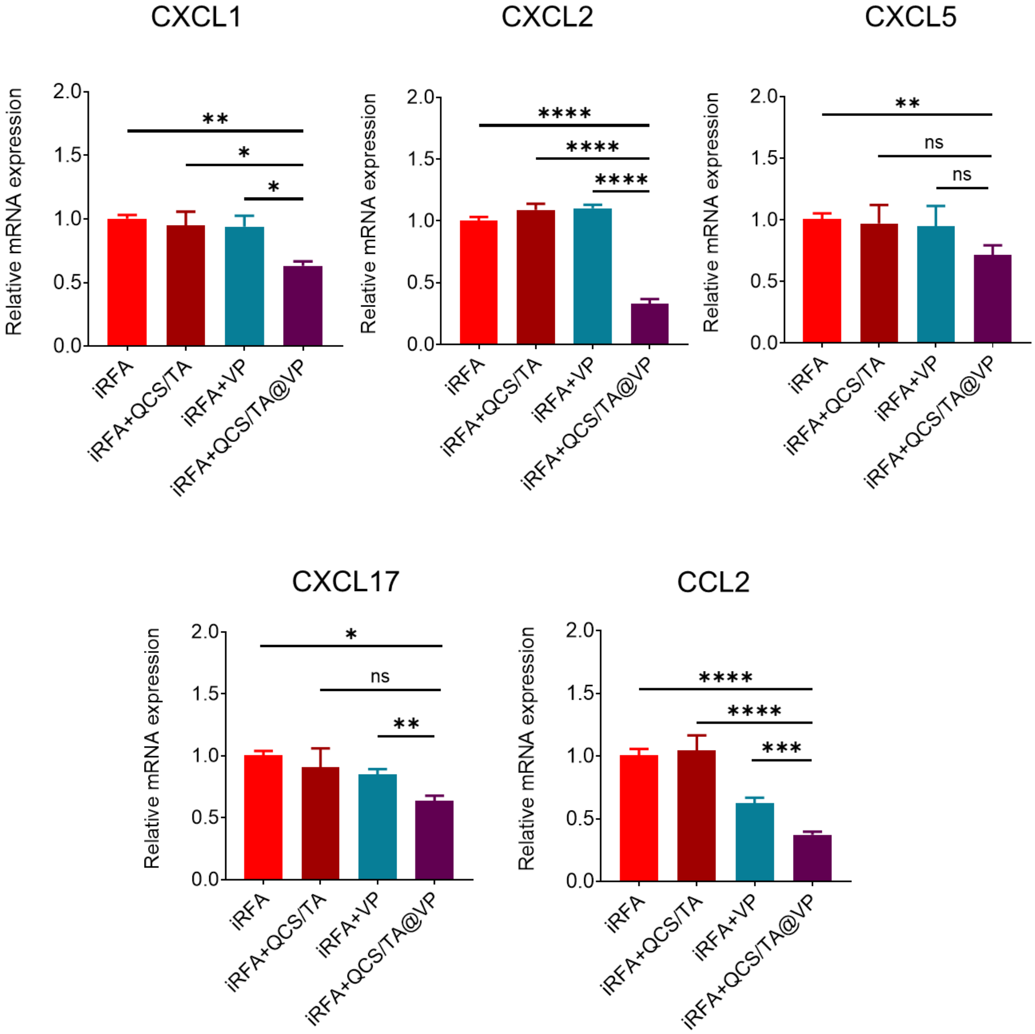

Figure. S16. qPCR analysis showing relative expression MDSCs-related cytokines following various treatments in residual cancer. *$P < 0.05$, **$P < 0.01$, ***$P < 0.001$, ****$P < 0.0001$

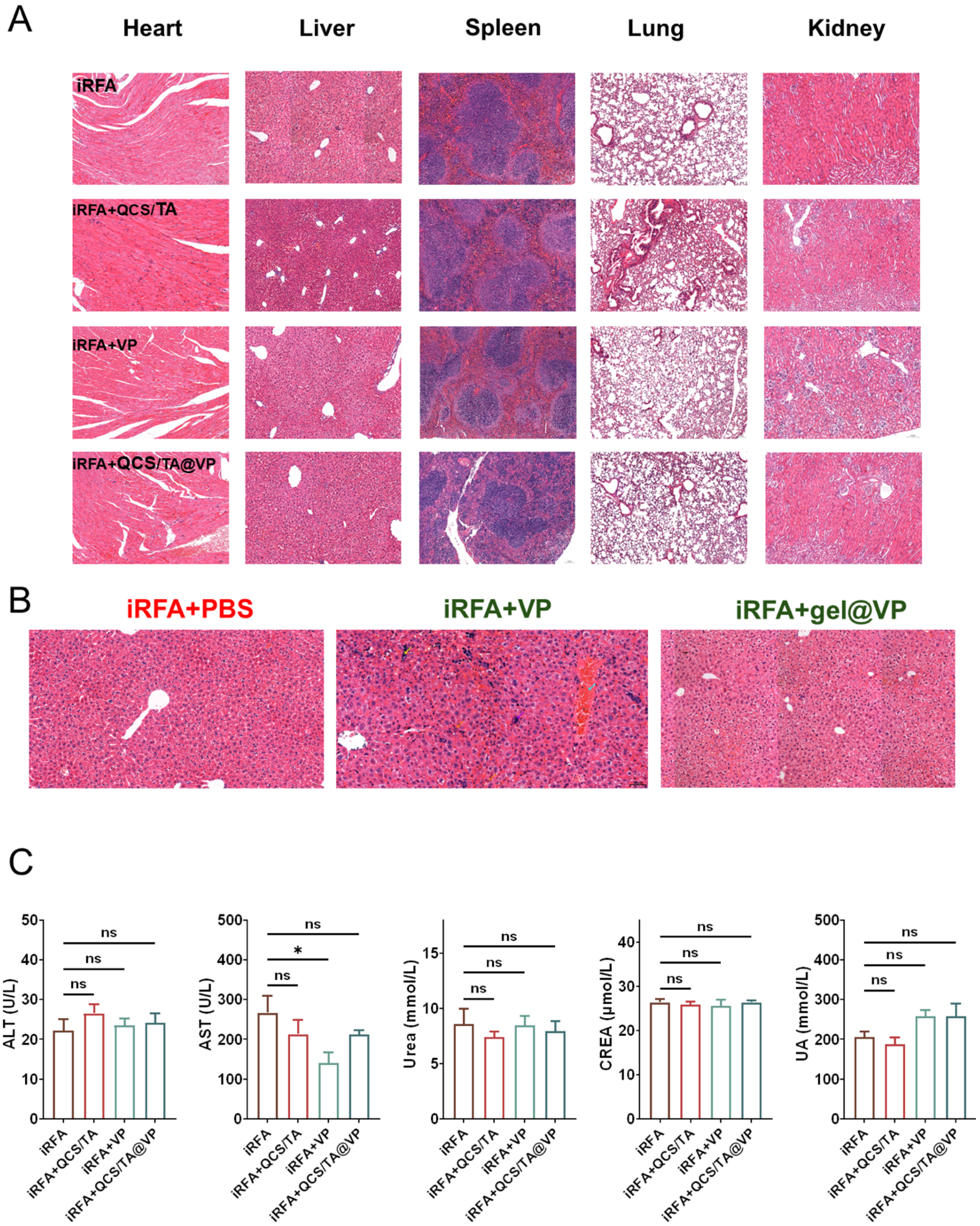
A
Heart
Liver
Spleen
Lung
Kidney
iRFA
iRFA+QCS/TA
iRFA+VP
iRFA+QCS/TA@VP
B
iRFA+PBS
iRFA+VP
iRFA+gel@VP
C
ALT (U/L)
AST (U/L)
Urea (mmol/L)
CREA (μmol/L)
UA (mmol/L)
ns
*
iRFA
iRFA+QCS/TA
iRFA+VP
iRFA+QCS/TA@VP

Figure. S17. (A) Representative HE staining images of heart, spleen, lung, and kidney in iRFA, iRFA + QCS/TA, iRFA + VP, and iRFA + QCS/TA@VP groups (scale bar: 100 μm). (B) Liver injury by H&E staining in different treatment groups. Scar bar: 500μm. (C) Blood analysis of Aspartate aminotransferase (AST), alanine aminotransferase (ALT), uric acid (UA), urea nitrogen (Urea), creatinine (CREA). *$P < 0.05$, **$P < 0.01$

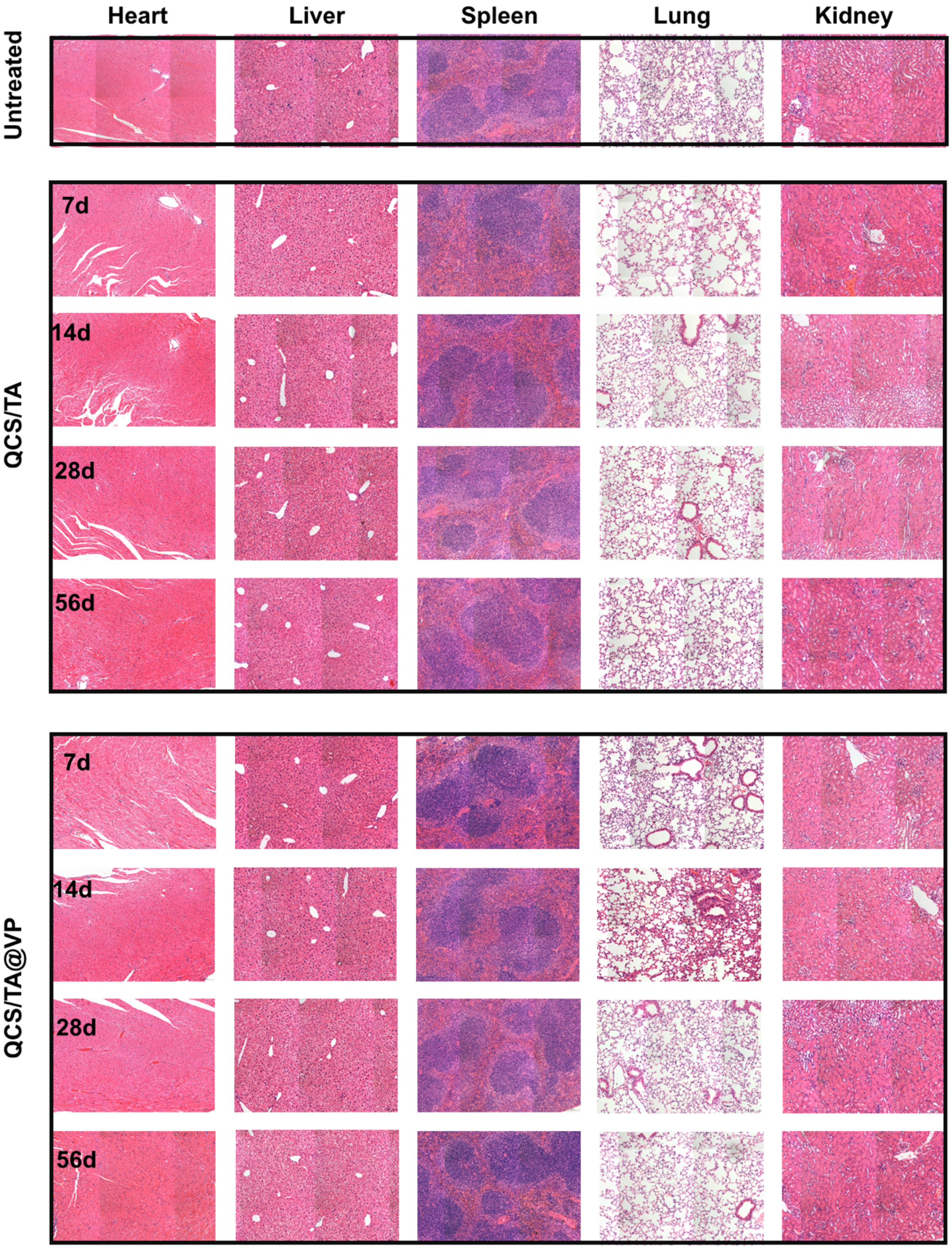
Heart
Liver
Spleen
Lung
Kidney
Untreated
QCS/TA
7d
14d
28d
56d
QCS/TA@VP
7d
14d
28d
56d

Figure. S18. Representative HE staining images of heart, spleen, lung, and kidney in Untreated, QCS/TA, and QCS/TA@VP groups (scale bar: 100 μm).

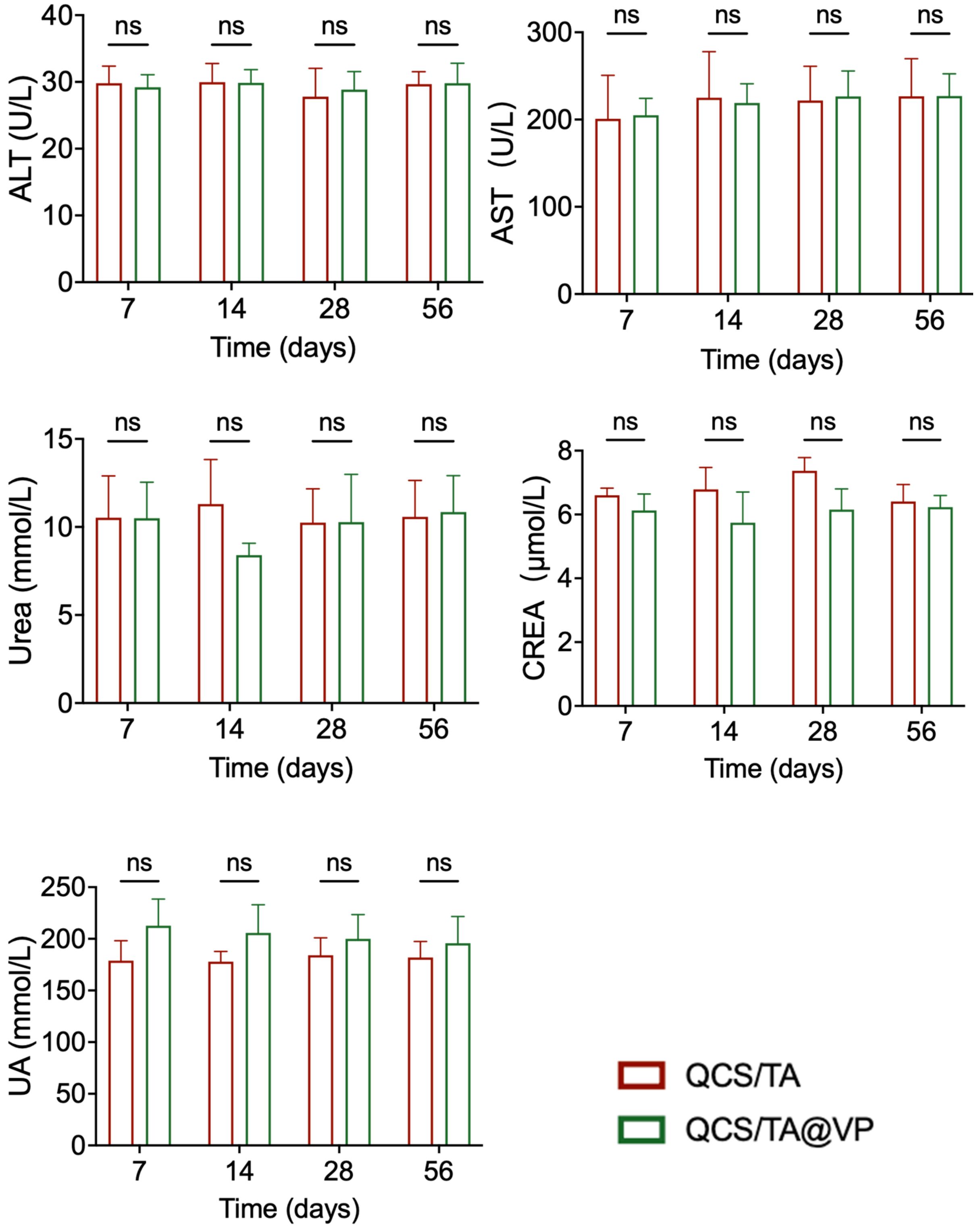
ALT (U/L)
AST (U/L)
Urea (mmol/L)
CREA (μmol/L)
UA (mmol/L)
ns
Time (days)
7
14
28
56
QCS/TA
QCS/TA@VP

Figure. S19. Blood analysis of Aspartate aminotransferase (AST), alanine aminotransferase (ALT), uric acid (UA), urea nitrogen (Urea), creatinine (CREA).

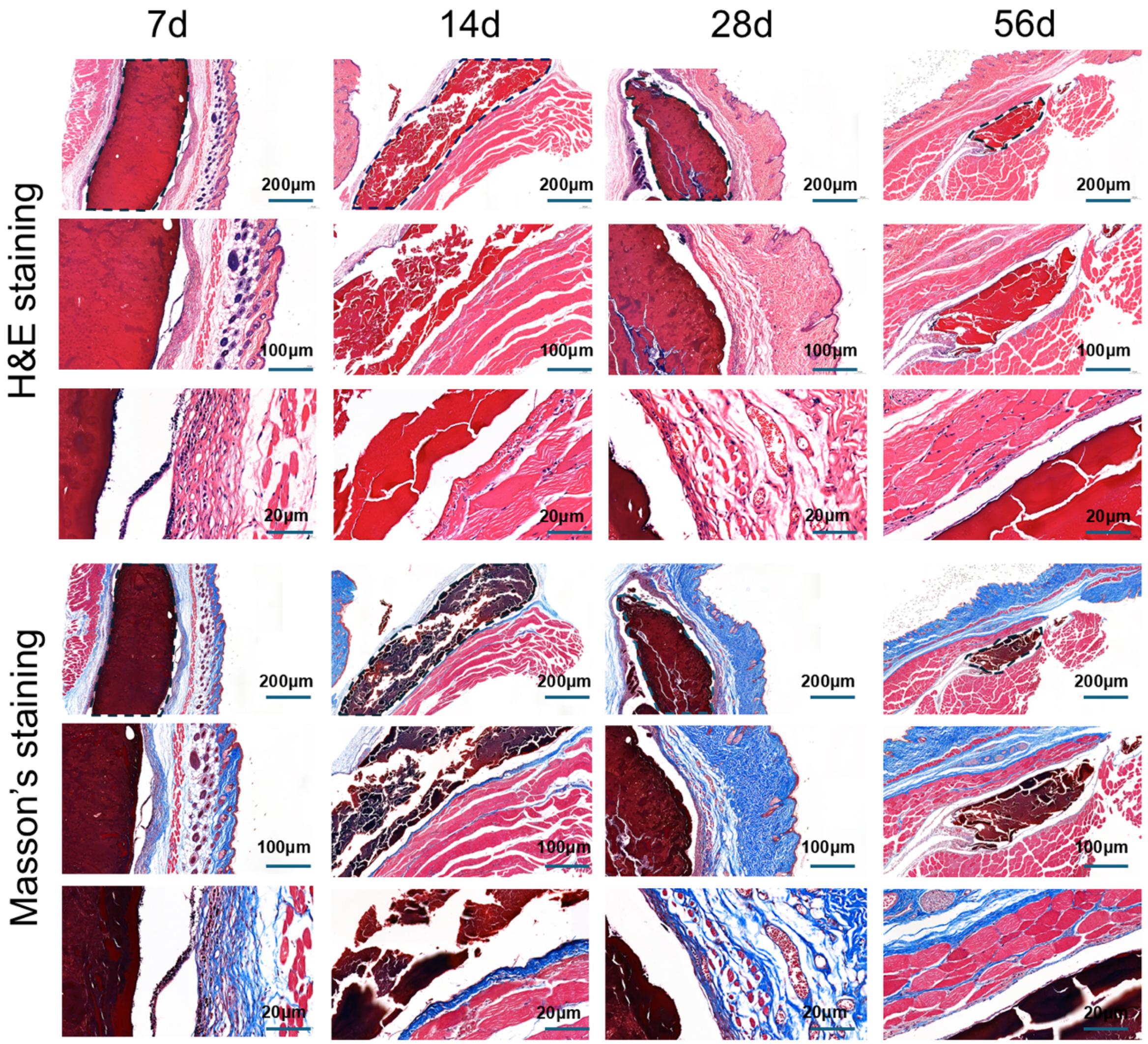

Figure.S20. Hematoxylin-eosin (H&E) staining (above) and Masson's staining (below) representative images of the tissues

Table S1. The Primer Sequence of Gene for RT-qPCR Analysis

Gene	Forward primer sequence（5'-3'）	Reverse primer sequence（5'-3'）
Cxcl1	GGCTGGGATTCACCTCAAGAACATC	TGAGTGTGGCTATGACTTCGGTTTG
CXCL1	GAACATCCAAAGTGTGAACGTGAAG	CTTAACTATGGGGGATGCAGGATTG
Cxcl2	CAGACAGAAGTCATAGCCACTCTCAAG	CAGTTAGCCTTGCCTTTGTTCAGTATC
CXCL2	CACACTCAAGAATGGGCAGAAAG	TTCTGGTCAGTTGGATTTGCCATTT
Cxcl5	TGTGTTTGCTTAACCGTAACTCCA	ACAGACCTCCTTCTGGTTTTTCAG
CXCL5	AGTAATCTGCAAGTGTTCGCCATAG	CTTTAGAAAAGGGGCTTCTGGATCA
Cxcl17	CCTCTCCCTTCCTTCTGTTGC	GCGACTTCCTGTGGTGCTTT
CXCL17	ACCGAGGCCAGGCTTCTA	GGCTCTCAGGAACCAATCTTT
Ccl2	TGATCCCAATGAGTAGGCTGGA	GACCTCTCTCTTGAGCTTGGTG
CCL2	CTCATAGCAGCCACCTTCATTCC	GAAGATCACAGCTTCTTTGGGACA
Cyr61	GAGTTACCAATGACAACCCAGAGTG	TTTCTTGGTCTTGCTGCATTTCTTG
CYR61	CTCGCCTTAGTCGTCACCC	CGCCGAAGTTGCATTCCAG
Ctgf	ACCCGAGTTACCAATGACAATACC	GGATGCACTTTTTGCCCTTCTTAAT
CTGF	CAGCATGGACGTTCGTCTG	AACCACGGTTTGGTCCTTGG
Gapdh	TGACCTCAACTACATGGTCTACA	CTTCCCATTCTCGGCCTTG
GAPDH	ACAACTTTGGTATCGTGGAAGG	GCCATCACGCCACAGTTTC
